# Functional screening of somatic mutant events in extranodal natural killer/T-cell lymphoma with adrenal involvement

**DOI:** 10.3389/fimmu.2025.1566794

**Published:** 2025-05-13

**Authors:** Luxin Zhang, Haifeng Gao, Shuang Ma, Xiaoming Fan, Huahang Guo, Man Sun, Shuang Wen, Tianqing Liu, Guanghai Yu, Xiaoying Yuan, Xiuhua Sun, Bo Fan

**Affiliations:** ^1^ Department of Urology, Second Affiliated Hospital of Dalian Medical University, Dalian, Liaoning, China; ^2^ Department of Urology, Central Hospital of Dalian, Dalian, Liaoning, China; ^3^ Institute of Cancer Stem Cell, Dalian Medical University, Dalian, Liaoning, China; ^4^ Department of Pathology, Central Hospital of Dalian, Dalian, Liaoning, China; ^5^ Department of Pathology, Dalian Friendship Hospital, Dalian, Liaoning, China; ^6^ Department of Emergency Medicine, Shengjing Hospital of China Medical University, Shenyang, Liaoning, China; ^7^ College of Humanities and Social Sciences, Dalian Medical University, Dalian, Liaoning, China; ^8^ Department of Oncology, Second Affiliated Hospital of Dalian Medical University, Dalian, Liaoning, China

**Keywords:** extranodal natural killer/T-cell lymphoma, adrenal, whole-genome sequencing, gene mutations, population-based study

## Abstract

**Background:**

Extranodal natural killer/T-cell lymphoma (ENKTL) involving the adrenal glands is extremely rare, and only a few cases have been reported. However, the genetic alterations, clinicopathological features and prognosis of these patients have not yet been fully elucidated.

**Methods:**

Profiling of tumor mutations in ENKTL patients with adrenal involvement was conducted by whole-genome sequencing, and the predisposing genes and driver mutation gene variants were verified through Sanger sequencing. Immunohistochemical analysis of markers for the diagnosis and tumor microenvironment competent were performed to identify histopathological features. In addition, we searched the Surveillance, Epidemiology, and End Results (SEER), PubMed, Embase, and Scopus databases to perform a population-based study to compare the prognosis between adrenocortical carcinoma (ACC) patients and adrenal ENKTL patients using Kaplan–Meier survival curves and log-rank tests and analyzed the prognostic factors affecting the overall survival (OS) of adrenal ENKTL patients via univariate and multivariate Cox regression analyses.

**Results:**

We screened 15892 somatic single-nucleotide variants (SNVs), 364 somatic insertions and deletions (INDELs), and four driver mutation genes, namely, TET2, STAT3, FAS, and TP53. In addition, immunohistochemical analysis revealed that tumor cells were positive for CD3, CD43, CD56, TIA1, granzyme B, CD2, CD4, and CD7. The immunohistochemistry for detecting components of the tumor microenvironment reveled the infiltration of tumor-associated macrophages (CD68, CD163) and tumor-associated fibroblasts (vimentin, SMA) in the tumor sample. According to our population-based analysis, Kaplan–Meier survival curves revealed that ENKTL patients with adrenal involvement had a significantly poorer prognosis than did patients with ACC (*p*<0.001), and chemotherapy was a significant prognostic factor for OS in ENKTL patients with adrenal involvement according to Cox multivariate analysis (hazard ratio = 0.318; *p*=0.027).

**Conclusions:**

The metastasis of ENKTL to the adrenal gland may be due to gene mutations caused by genetic variations, which may provide new therapeutic targets for this disease. The prognosis of adrenal ENKTL patients is markedly worse than that of ACC patients, and chemotherapy may serve as an independent factor of OS in adrenal ENKTL patients. However, our findings still need to be validated in additional studies.

## 1 Introduction

Extranodal natural killer/T-cell lymphoma (ENKTL) is a particularly aggressive subtype of non-Hodgkin lymphoma that is intimately related to infection with Epstein–Barr virus (EBV) (1). The prevalence of ENKTL is significantly greater in Central and South America and Asia than in the United States and Europe (2, 3). Approximately 80% of ENKTL cases develop in the upper respiratory tract, which includes the nasal cavity, nasopharynx, paranasal sinuses, and palate, with the nasal cavity being the most frequent site; approximately 20% of cases develop in extranasal regions, including the skin, gastrointestinal tract, testes and salivary glands (1, 4). The process of tumorigenesis in ENKTL has multiple stages. There are two widely accepted assumptions about these multistage processes. One hypothesis is that NK/T cells can be infected by EBV and assisted by EBV oncogenes such as EBNA1 and LMP1 to proliferate and avoid apoptosis. In long-term disease processes, cumulative variations in driver genes, such as JAK3 and DDX3X, may contribute to the progression of ENKTL. The other hypothesis is that EBV-infected cells can accumulate mutations due to senescence and chronic exposure to environmental stressors (5, 6). ENKTL always exhibits an invasive course and poor clinical outcome, especially in the presence of extranasal metastases, with a complete remission rate of only approximately 30% and a median survival of 4.3 months (7).

Patients with ENKTL concomitant with adrenal involvement are extremely rare; therefore, the clinicopathological features and genetic characteristics of these patients have not been systematically described. In this study, we reported an eight-year episode of an ENKTL patient with adrenal and multiple site involvement who achieved a complete response (CR) after undergoing surgery, chemotherapy and immunotherapy and remained in good general condition after 101 months of follow-up. Next, we evaluated the genetic alterations of the patient by conducting next-generation sequencing (NGS). Additionally, we reviewed published cases of ENKTL with subsequent adrenal involvement, compared the prognosis of ENKTL patients with adrenal involvement and adrenocortical carcinoma (ACC) patients and explored the risk factors for the prognosis of adrenal ENKTL patients.

## 2 Materials and methods

### 2.1 Clinical data

We obtained the patient’s clinical data from the Dalian Central Hospital and the Second Affiliated Hospital of Dalian Medical University. The Ethics Committee of Dalian Central Hospital (ethical approval number: YN2024-029-01) and the Second Affiliated Hospital of Dalian Medical University (ethical approval number: 2023273) approved the research respectively, and the patient provided informed consent. The research complied with the Declaration of Helsinki.

### 2.2 Immunohistochemical analysis

The formalin-fixed tissues were embedded in paraffin and subsequently cut into 5-µm sections. The sections were dewaxed in xylene and hydrated in ethanol, and antigen repair was performed by placing the slides in sodium citrate buffer and heating them at 95°C for 20 minutes. The slides were incubated in 0.5% hydrogen peroxide for 20 min to block endogenous peroxidase activity, blocked with goat serum for 1 h, and incubated overnight at 4°C with the primary antibodies. **Supplementary Table S1** lists the antibodies used in this study. Following the manufacturer’s instructions, the slides were incubated with an avidin-biotin kit. The slides were developed in the chromogen 3,30-diaminobenzidine for 5 minutes at nearly 24°C, and hematoxylin was used for counterstaining for 30 seconds. Microscopy (Leica, Wetzlar, Germany) was used to observe the slides. We detected EBV-encoded small RNAs (EBERs) using an EBV Probe *In Situ* Hybridization Kit (Zhongshan Golden Bridge Biotechnology Co. Ltd., Beijing, China).

### 2.3 Population-based study

#### 2.3.1 Data resources and study population

We searched the SEER database (https://seer.cancer.gov/) on June 2023, which consists of 17 registries covering more than 26.5% of the United States population between 2000 and 2020, for information on adrenocortical carcinoma (ACC) patients. Patients with an International Classification of Disease (ICD)-O-3 code of 8370 with malignant behavior were screened. We included a total of 1771 ACC patients in our study after excluding patients with a primary site in the medulla of the adrenal gland, missing histological confirmation or unknown survival time. Patients whose ICD-O-3 code was 9719 and whose primary site was the adrenal gland were screened; 2 ENKTL patients with adrenal involvement were included in our study. Moreover, we searched the PubMed, Embase and Scopus databases using the keywords “natural killer/T cell lymphoma”, “NK/T cell lymphoma” and “adrenal” for ENKTL patients with adrenal involvement up to April 2025. Together with the one patient we reported, a total of 39 patients were included in our study, and the detailed selection flowchart is shown in **Supplementary Figure S1**. Patient characteristics and survival information, including age, sex, race, primary site, Ann Arbor stage, absence or presence of B symptoms, laterality, tumor size, surgery, radiotherapy and chemotherapy, were summarized. The main endpoint was overall survival (OS).

#### 2.3.2 Statistical analyses

Count data were transformed into categorical variables, and comparisons between ACC patients and ENKTL patients with adrenal involvement were performed using Fisher’s exact probability or Pearson’s chi-square method using SPSS version 26.0 (SPSS Inc., Chicago, IL, USA). We used the ‘survival’ package in R software (version 4.3.1, http://www.R-project.org/) to conduct log-rank tests and plot Kaplan–Meier survival curves. Univariate and multivariate Cox regression analyses were performed to calculate hazard ratios (HRs) and 95% confidence intervals (CIs). A p value <0.05 (two-sided) was considered to indicate statistical significance.

### 2.4 Whole-genome sequencing

#### 2.4.1 DNA extraction

The FFPE tumor specimens and matched normal specimens were subjected to DNA extraction with a GeneRead DNA FFPE Kit (Qiagen, Germany) according to the manufacturer’s instructions. The extracted specimens were subjected to whole-genome sequencing. We evaluated the DNA quality through agarose gel electrophoresis and a Qubit^®^ DNA Assay Kit in a Qubit^®^ 3.0 Fluorometer (Invitrogen, USA). We performed library construction using DNA samples with concentrations above 20 ng/µL and a total quantity above 0.2 µg.

#### 2.4.2 Library preparation and sequencing

We generated the sequencing libraries using the NEBNext^®^ Ultra™ DNA Library Preparation Kit (NEB, USA) following the manufacturer’s instructions and added an index code to each sample. After the genomic DNA samples were fragmented into 350 bp fragments by sonication, the DNA fragments were polished, a-tailed and ligated to full-length adapters, followed by further PCR amplification. We purified the PCR products with an AMPure XP system (Beckman Coulter, Beverly, USA) and then amplified them using a Qubit^®^ 3.0 Fluorometer (Invitrogen, USA) to detect DNA concentrations and an NGS3K/Caliper to analyze the size distribution of the libraries and quantify them by real-time PCR (3 nM). We clustered the index-coded samples on the cBot Cluster Generation System using the Illumina PE Cluster Kit (Illumina, USA). We then sequenced the DNA library on the Illumina platform to produce 150 bp paired-end reads.

#### 2.4.3 Quality control

Raw data were filtered by removing single-end sequencing reads with adapters, more than 10% of unidentifiable base information, or more than 50% of the read length with low quality (fewer than 5 bases). The sequencing error rate for each base position was than 1%, and the quality of the sequencing data was distributed at a Q30 of no less than 80%.

#### 2.4.4 Bioinformatics analysis

We aligned the validated sequencing data with the reference genome (GRCh37/hg19/GRCh38) by BWA (8) to obtain the initial alignment results in the BAM format. The results were then ranked using SAMtools (8), and Sambamba was used to mark duplicate reads. Finally, the coverage and depth statistics of the marked duplicate reads were calculated.

#### 2.4.5 Variant detection, somatic mutation calling and functional annotation

Based on the initial results (BAM files), SAMtools (9) was used to identify and count the number of single nucleotide polymorphism (SNPs), copy number variations (CNVs) increases and decreases were detected using Control-FREEC (10), which calculates the number of different types of CNV events. The software Lumpy (11) was used to detect structural variations (SVs) and count the number of different types of SVs. MuTect (12), Strelka (13), Control-FREEC (10) and Lumpy (11) were used to detect somatic single-nucleotide variants (SNVs), insertions and deletions (INDELs), CNVs and SVs. Functional annotation of the detected gene variants was performed using ANNOVAR (14).

#### 2.4.6 Identification of potential predisposing genes and driver mutations

We used SAMtools (9) to identify germline mutations (SNPs and INDELs) in the normal tissues of patients from the Cancer Gene Census (CGC) database, Familial Cancer Database, and intOGen database and the reported genes summarized in the Nature literature, and the detected mutations were screened for potential predisposing genes. We examined somatic mutations (SNVs and INDELs) in tumor specimens using MuTect software and then screened for known driver genes by using Bert Vogelstein (15), significant mutated genes (SMGs) (16) and the CGC database (17).

#### 2.4.7 Analysis of tumor purity, tumor ploidy and targeted drug prediction

We used ABSOLUTE to calculate the purity (the proportion of tumor cells to total cells), ploidy (the average copy number of the sample) and cancer DNA fraction (the proportion of tumor DNA to total DNA) of the tumor sample to guarantee analysis quality (18). To identify potentially actionable mutations, tumor-derived genetic alterations were cross-referenced with the NovoDrug knowledgebase for targeted therapy prediction. This comprehensive resource combines validated therapeutic targets from authoritative references including DrugBank, the Pharmacogenomics Knowledge Base (PharmGKB), My Cancer Genome, the Food and Drug Administration (FDA) and Kyoto Encyclopedia of Genes and Genomes (KEGG) databases, supplemented with investigational agents from clinical trial registries like ClinicalTrials.gov. Concurrently, tumor-specific somatic variants were analyzed against the NovoDR database to detect putative resistance markers, providing clinically relevant insights for treatment selection and outcome evaluation (19).

#### 2.4.8 Sanger sequencing

To confirm that the predisposing and driver genes containing mutant bases were detected by NGS, Sanger sequencing was performed. Genomic DNA was extracted according to the manufacturer’s instructions, after which polymerase chain reaction (PCR) was performed. After purification of the PCR products by agarose electrophoresis, the PCR products were sequenced on an Applied Biosystems™ 3730xl sequencer.

#### 2.4.9 Gene mutation analysis across cancers

We used the cBioPortal database (https://www.cbioportal.org/, accessed on 14 August 2023) to analyze the mutant genes confirmed by Sanger sequencing across cancers.

#### 2.4.10 Structure of protein–protein interaction networks

The protein–protein interaction (PPI) network of the genes confirmed by Sanger sequencing was constructed through the Search Tool for the Retrieval of Interacting Genes/Proteins (STRING) database with a minimum required interaction score of 0.15 and a maximum number of interactors of 50. Then, the PPI network was visualized using Cytoscape software (version 3.9.1).

#### 2.4.11 GO and KEGG pathway enrichment analyses

The biological functions of the node genes in the PPI network were analyzed through Gene Ontology (GO) (20) and KEGG (21) pathway enrichment analyses, setting a statistically significant difference threshold of q value < 0.05 using the “clusterProfiler” R package (version 4.3.1), and the top 20 genes of each analysis were visualized with the “GOplot” R package.

## 3 Results

### 3.1 Case presentation

A 57-year-old female patient was admitted to our hospital due to recurrent yellow discharge in the left nasal region and fixed-site headaches secondary to nasal obstruction after upper respiratory tract infections for two years, which were aggravated for two weeks. She underwent surgical resection of a right nasal hemangioma eight years prior. Physical examination revealed right deviation of the nasal septum, right nasal mucosa erosion, crust on the right middle nasal passage, right nasal stenosis, and crust and discharge in the left nasal cavity with unclear nasal structure. Computed tomography (CT) of the paranasal sinus revealed bilateral maxillary and ethmoid sinusitis, thickened right nasal mucosa, partial absence of the middle and inferior turbinate of the right nasal cavity, a growing mass of soft tissue density in the left nasal cavity and left nasal cavity stenosis. Massive amounts of necrotic tissue were removed from the left nasal cavity, and the diagnosis of ENKTL was confirmed by pathological examination of the discharge of the left nasal cavity combined with immunohistochemical results and *in situ* hybridoma with EBERs. Beginning in October 2016, the patient was treated with 54 Gray local nasal radiotherapy (30 cycles of 1.8 Gray) for 2 months, which resulted in unconfirmed complete remission (uCR), but she did not undergo regular reexamination after radiotherapy.

**Figure 1:**
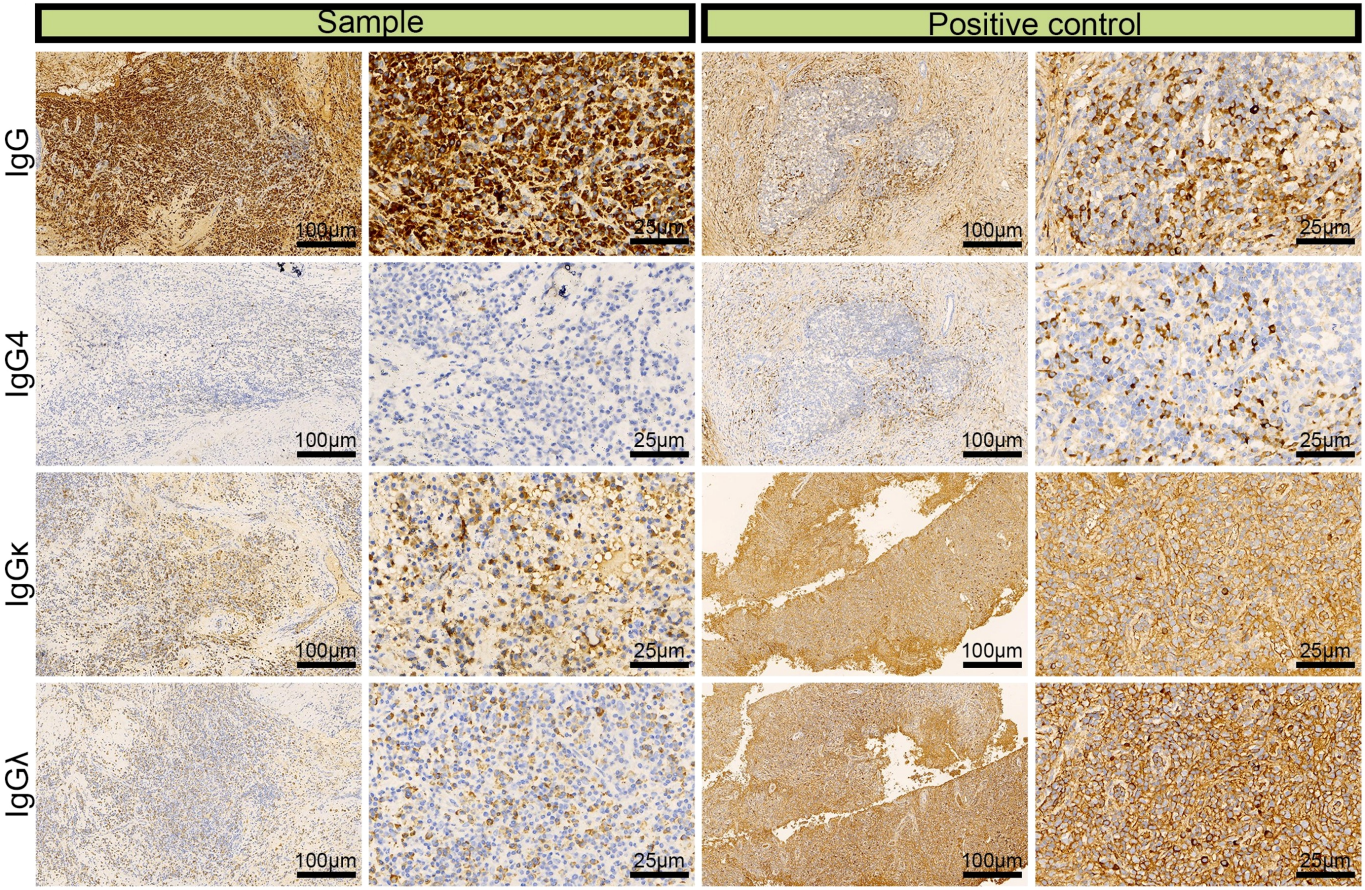
Immunohistochemical images of IgG, lgG4, lgGκ, and lgGλ in patients with left nasal sinusitis (scale bar: 25 μm).

The patient experienced nasal obstruction for one month and was admitted to the hospital again after four years. Physical examination revealed nasal septum perforation, hypertrophy of the middle and inferior turbinate in the right nasal cavity with sticky discharge on the surface, adhesion of the left nasal cavity and invisible middle nasal meatus. Paranasal sinus CT revealed bilateral maxillary and ethmoidal sinuses filled with soft tissue and a decrease or disappearance of the partial cavity, an enlarged maxillary sinus orifice, partial absence of bilateral turbinate and hyperplasia of soft tissue in the nasal cavity. A nasopharyngoscope revealed a fragile pale mass in the left nasal cavity with a rough surface and unclear nasal structures, and no mass was found in the unobstructed right nasal cavity. Due to the expansive growth of the mass in the left nasal cavity and secondary bone destruction of the lateral nasal wall, the patient underwent endoscopic mass excision of the left nasal cavity. Pathology examination of a 0.4 cm mass resected from the ethmoid sinus and a 0.8 cm mass resected from the maxillary sinus demonstrated a composition of inflammatory granulation tissue and bone tissue with many plasma cells infiltrating the mesenchyme. Based on these findings combined with the immunohistochemical results of IgG (+), IgG4 (–), κ (+), and λ (+) (**Figure 1**), the patient was diagnosed with left nasal sinusitis rather than ENKTL recurrence.

**Figure 2:**
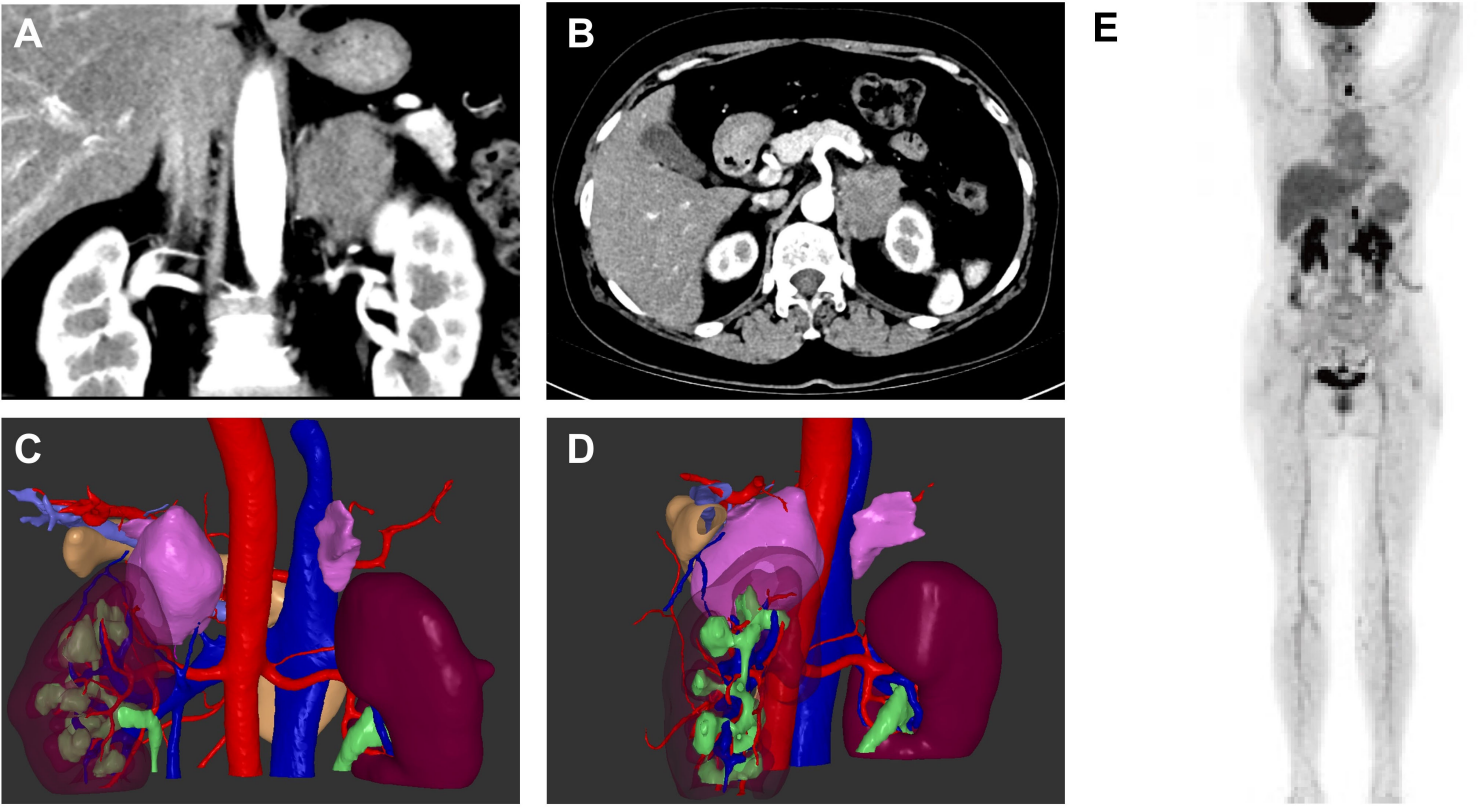
Enhanced computed tomography images of the left adrenal mass in sagittal and coronal views **(A, B)** and three-dimensional construction images **(C, D)**. ^18^F-FDG PET/CT of the patient **(E)**.

One year later, the patient was referred to our hospital for a third visit and complained of left chest and back pain unrelated to breathing for four days. Chest CT and contrast-enhanced CT of the adrenal glands revealed a left adrenal mass with a diameter of 4.7 cm that showed heterogeneous enhancement, as shown by the three-dimensional structure in **Figure 2**. We ruled out primary adrenal malignancy owing to normal adrenal hormone levels as follows: epinephrine <0.14 nmol/L (normal 3.24–18 nmol/L), norepinephrine 8.52 nmol/L (normal 0.41-4.43 nmol/L), and dopamine 0.10 nmol/L (normal <0.2 nmol/L). However, the CT findings were indicative of adrenal malignancy; therefore, laparoscopic resection of the tumor was performed. The diagnosis of ENKTL was confirmed by postoperative pathology and immunohistochemistry.

To help assign the stage and tailor the treatment plan, the patient underwent 18F-fluoro-2-deoxy-D-glucose positron emission tomography-CT examination, as shown in **Figure 2E**, revealing homogeneous increases in FDG uptake in the mucosa of the nasal tract (maximal standardized uptake value (SUVmax), 3.5-3.7), the left tonsil (SUVmax, 6.5), the left lobe of the thyroid (SUVmax, 23.1), the soft tissue in the left adrenal region (SUVmax, 16.5) and the left supraclavicular lymph node (SUVmax, 3.1). Bone marrow aspirate smears showed no evidence of malignant lymphoma, and the patient had no type B symptoms, such as fever, chills, or night sweats. According to the results, the patient was diagnosed with extranodal natural killer/T-cell lymphoma, nasal type (ENKTL-NT), stage IV. The patient underwent chemotherapy using the P-GEMOX regimen, which included gemcitabine (2 g, Day 1), oxaliplatin (150 mg, Day 1) and pegaspargase (3750 U, Day 1), for a cycle of 21 days. Radiological imaging was used to evaluate the therapeutic effect. After two cycles of chemotherapy, the patient suffered from significant general weakness, nausea, decreased appetite and reduced food intake. Laboratory findings revealed increased alanine aminotransferase (112.86 U/L) and aspartate aminotransferase (59.59 U/L) levels, which was considered to indicate liver damage caused by chemotherapy; therefore, the dose of gemcitabine was reduced to 1.6 g beginning in the third cycle. The patient achieved CR after 6 cycles. Then, PD-1 monoclonal antibody (sintilimab, 200 mg, Day 1) was regularly administered every three weeks. The patient achieved CR after 15 cycles of treatment and continued regular therapy with sintilimab. As of April 2025, the patient had undergone 30 cycles of sintilimab treatment with no signs of recurrence and was in good general condition.

### 3.2 Pathological features of adrenal ENKTL

#### 3.2.1 Histopathological presentation

The left adrenal surgical specimen measured 4.5*5.0 cm with a complete capsule and a yellow to brown cut surface. HE staining revealed a destroyed adrenal structure with abundant diffusely distributed malignant tumor cells of a medium-large size with an irregular nucleus; light cytoplasmic staining; and aggressively growing tumor cells that invaded thick-walled blood vessels, enveloped the nerves, and infiltrated the adrenal tissue, exhibiting multiple annular foci.

#### 3.2.2 Immunohistochemical profile for diagnosis

**Figure 3:**
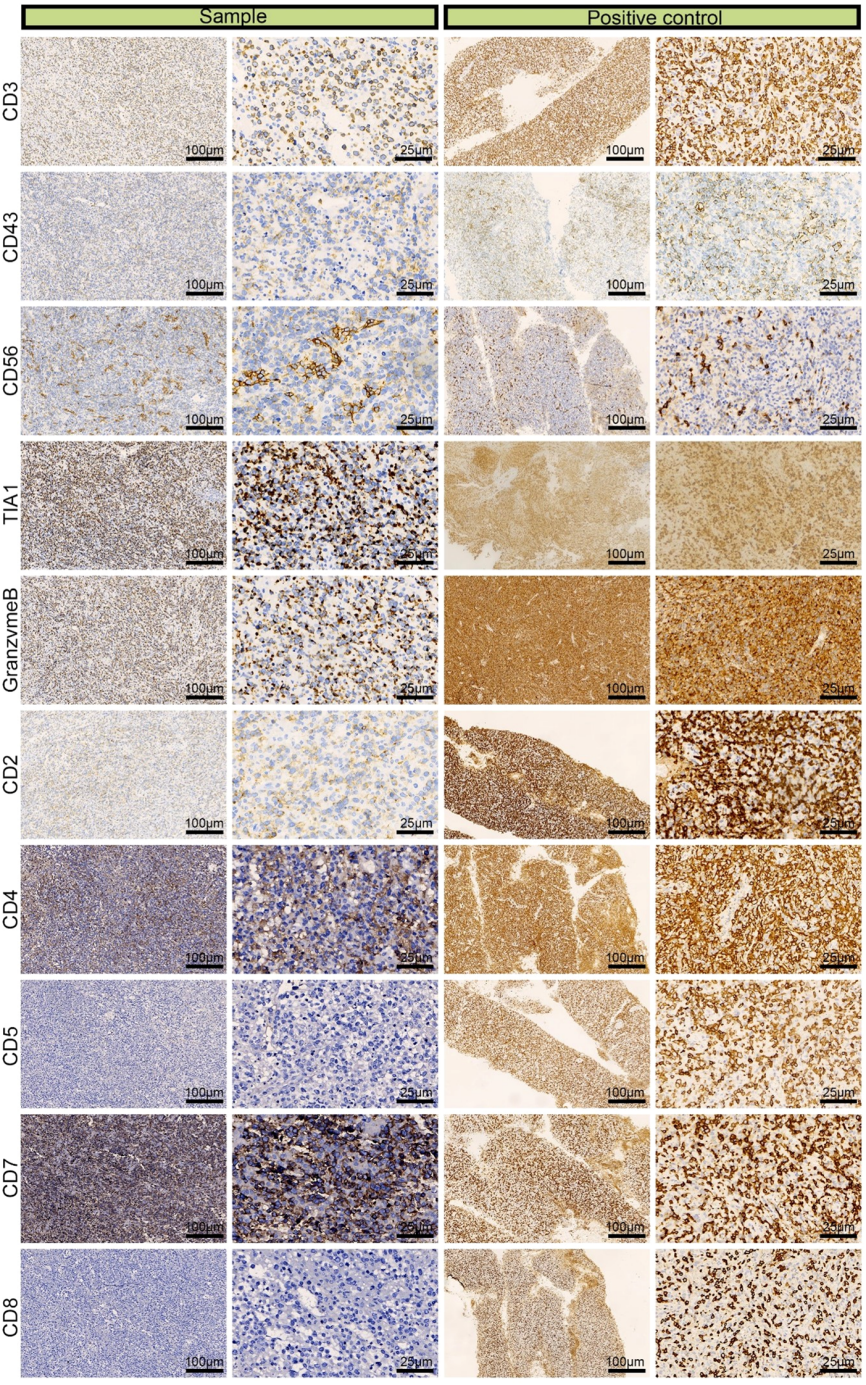
Immunohistochemical images of CD3 (+), CD43 (+), CD56 (partial +), TIA (+), granzyme B (+), CD2 (+), CD4 (partial +), CD5 (–), CD7 (+), and CD8 (–) from a left adrenal sample obtained by laparoscopic resection.

**Figure 4:**
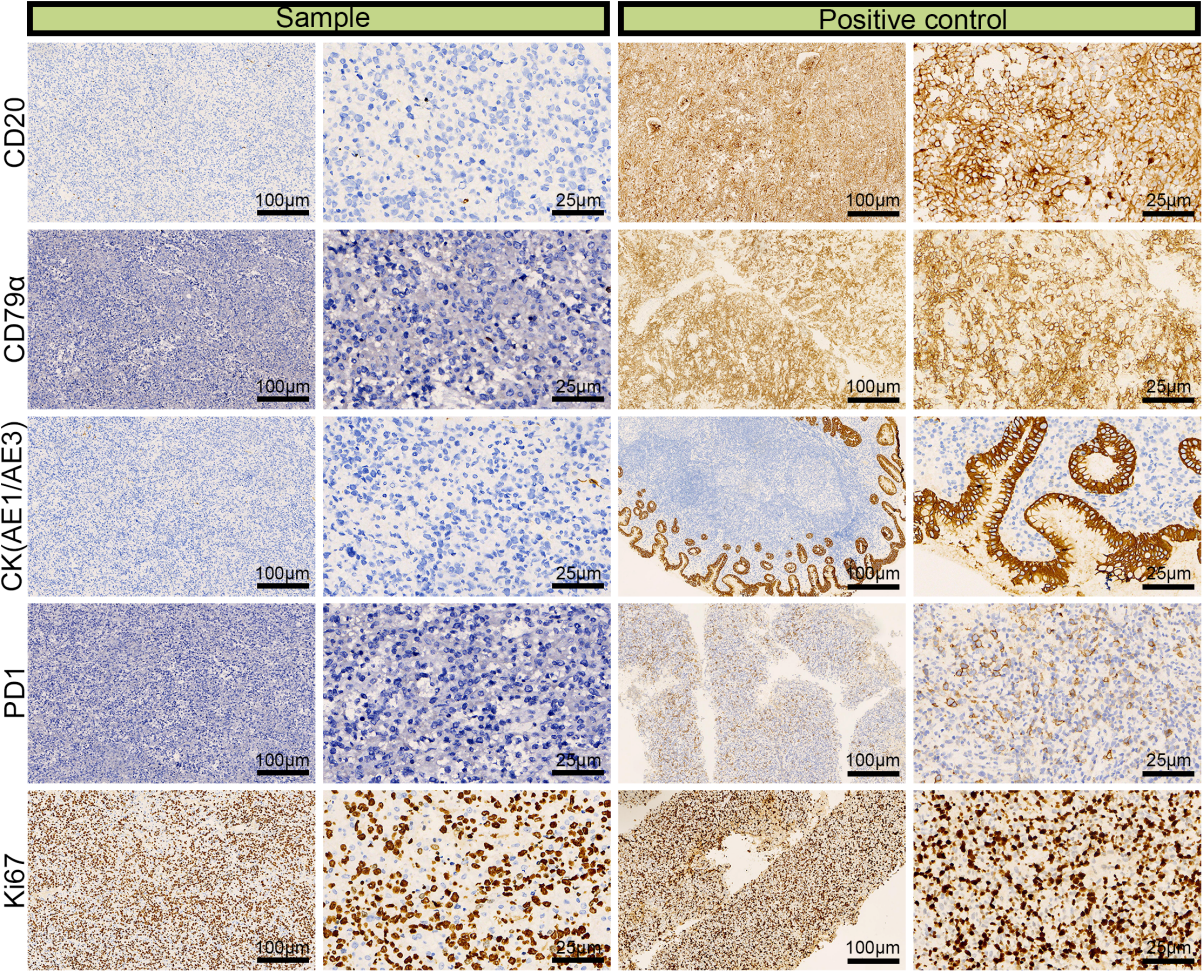
Immunohistochemical images for CD20 (–), CD79α (–), CK (AE1/AE3) (–), and the 90% labeling index of Ki-67 in a left adrenal sample obtained via laparoscopic resection.

We performed IHC analysis, and the following results contributed to the diagnosis of ENKTL: CD3 (+), CD43 (+), CD56 (partial +), TIA (+), granzyme B (+), CD2 (+), CD4 (partial +), CD5 (–), CD7 (+), and CD8 (–) (**Figure 3**). Moreover, we excluded the diagnosis of B-cell lymphoma based on the CD20 (–) and CD79α (–) results and epithelial-origin tumors based on the CK(AE1/AE3) (–) result. The Ki-67 labeling index was high (90%), as shown in **Figure 4**. *In situ* hybridization demonstrated the presence of EBER.

#### 3.2.3 Immunohistochemistry for detecting components of the tumor microenvironment

**Figure 5:**
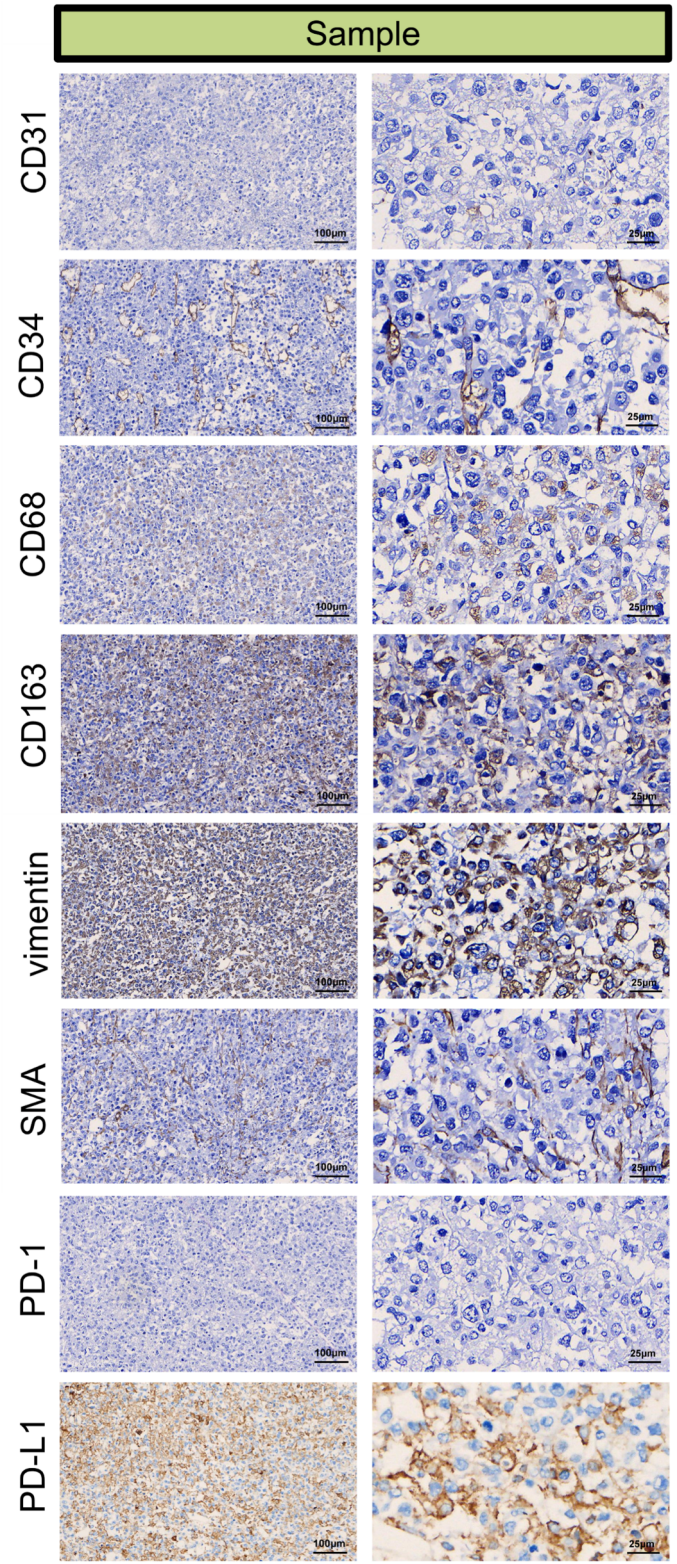
Immunohistochemical microscopic images of CD31 (partial +), CD34 (+), CD68 (+), CD163 (+), vimentin (+), SMA (+), PD-1 (–), PD-L1 (+) for the tumor micro-environment analysis in the tumor sample.

For immunohistochemistry to detect components of the tumor microenvironment, we analyzed the distributions of PD-L1, markers of vascular endothelial cells (CD31 and CD34), markers of TAMs (Tumor-associated macrophages, CD163 and CD68), markers of tumor-associated fibroblasts (vimentin, SMA) in the tumor tissue. As shown in **Figure 5**, positive staining of PD-L1, CD31 (partial +), CD34, CD68, CD163, vimentin and SMA detected in the tumor tissue, indicating that vascular endothelial cells, TAMs and fibroblasts may participate in the pathogenesis of ENKTL.

### 3.3 Population-based study

#### 3.3.1 Patient clinical and demographic characteristics

**Figure 6:**
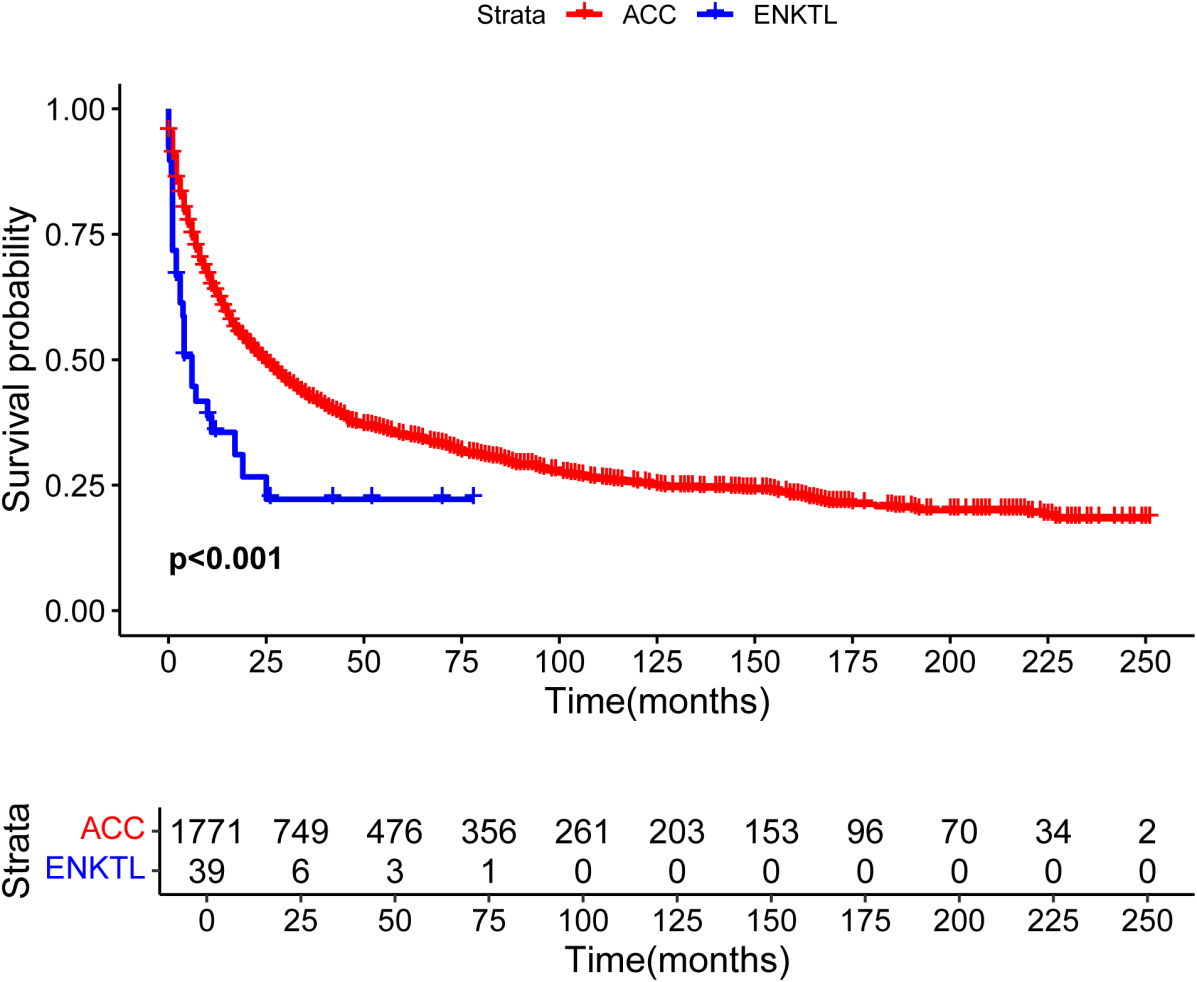
Overall survival curves of patients with extranodal natural killer/T-cell lymphoma (ENKTL) and adrenocortical carcinoma (ACC).

Until April 2025, 1771 ACC patients in the SEER database and 39 ENKTL patients with adrenal involvement were included in our study. Compared with those of ACC patients, the clinical and demographic characteristics of ENKTL patients with adrenal involvement, except for age and radiotherapy, were highly heterogeneous. The detailed clinical and demographic characteristics are presented in **Table 1** and **Supplementary Table S2**. As shown in the Kaplan–Meier survival plot in **Figure 6**, ENKTL patients with adrenal involvement exhibited significantly.

**Table 1:** Baseline characteristics of patients with adrenal extra-nodal natural killer/T -cell lymphoma (ENKTL) and adrenocortical carcinoma (ACC).

	ENKTL n=39 (%)	ACC n=1771 (%)	*p* value
Age			
<60	26 (66.7%)	1069 (60.4%)	0.528
>=60	13 (33.3%)	702 (39.6%)	
Sex			
Male	28 (71.8%)	703 (39.7%)	<0.001
Female	11 (28.2%)	1068 (60.3%)	
Race			
White	6 (15.4%)	1484 (83.8%)	<0.001
Black	0 (0.0%)	145 (8.2%)	
Asian or Pacific Islander	30 (76.9%)	124 (7.0%)	
Others	3 (7.7%)	18 (1.0%)	
Laterality			
Left	6 (15.4%)	926 (52.3%)	<0.001
Right	2 (5.1%)	786 (44.4%)	
Both	20 (51.3%)	8 (0.5%)	
Others	11 (28.2%)	51 (2.9%)	
Tumor size			
<80	8 (20.5%)	395 (22.3%)	<0.001
>=80	9 (23.1%)	940 (53.1%)	
Unknown	22 (56.4%)	436 (24.6%)	
Surgery			
No/unknown	33 (84.6%)	470 (26.5%)	<0.001
Yes	6 (15.4%)	1301 (73.5%)	
Radiotherapy			
No/unknown	36 (92.3%)	1498 (84.6%)	0.271
Yes	3 (7.7%)	273 (15.4%)	
Chemotherapy			
No/unknown	7 (17.9%)	1059 (59.8%)	<0.001
Yes	32 (82.1%)	712 (40.2%)	

#### 3.3.2 Identifying prognostic factors for ENKTL patients with adrenal involvement

**Figure 7:**
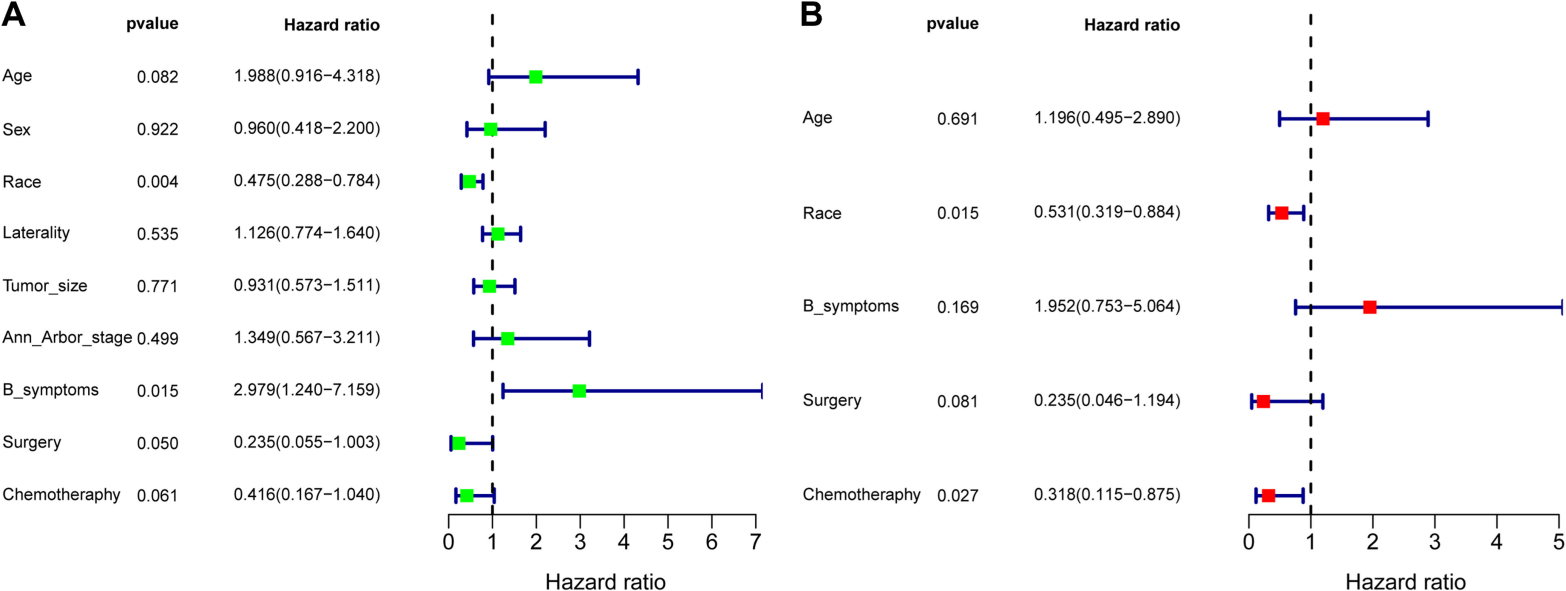
Forest plot of the univariate regression analysis **(A)** and multivariate regression analysis **(B)** revealing the relationships between different prognostic factors and overall survival in extranodal natural killer/T-cell lymphoma (ENKTL) patients (n=39). The tetragonal diamonds represent the hazard ratios (HRs), and the horizontal line crossing the diamonds represents the 95% confidence intervals (CIs). The data were analyzed by a Cox proportional hazards model and are shown as HRs and 95% CIs.

To determine the prognostic factors influencing the OS of ENKTL patients with adrenal involvement, we conducted univariate and multivariate Cox regression analyses. As shown in the forest plot in **Figure 7A**, race (*p*=0.004) and B symptoms (*p*=0.015) were identified as factors related to prognosis in the univariate Cox regression analysis. The forest plot of the multivariate Cox regression analysis in **Figure 7B** shows that race (*p*=0.015) and chemotherapy (*p*=0.027) was an independent protective factor.

### 3.4 Whole-genome sequencing study

#### 3.4.1 Identification of SNPs and INDELs

**Figure 8:**
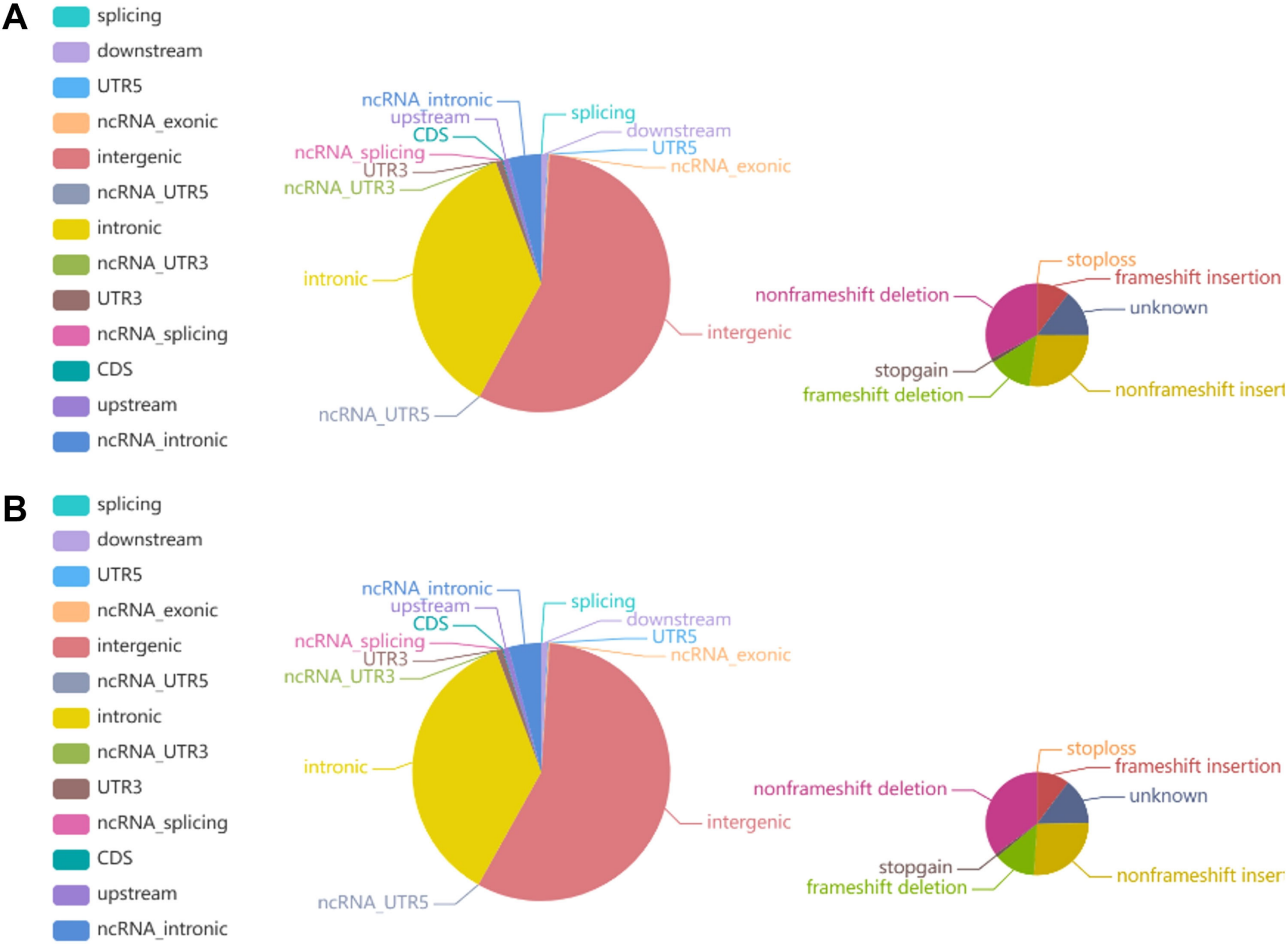
Pie chart representing the distribution of the number of INDELs. **(A)** The left and right images show the number of INDELs in different regions of the genome and in coding regions in tumor tissue, respectively. **(B)** The left and right images show the number of INDELs in different regions of the genome and the coding regions in normal tissue, respectively.

The average Q30 of the sequencing samples was 91.76%, and the average error rate was 0.03%, indicating that the sequencing data were of good quality and met the analysis requirements. We sequenced 263,986,447 and 228,437,421 read pairs in the two tumor specimens and 243,399,667 and 212,257,867 read pairs in the two matched normal specimens. A total of 3,400,005 and 3,504,202 SNPs were detected in the tumor specimens and matched normal specimens, respectively. The transformation/inversion ratio of 2.08 reflects the accuracy of the SNP dataset. A total of 497,401 and 535,209 INDELs were verified in the tumor specimens and matched normal specimens, respectively. The details of the INDELs are shown in **Figure 8** and **Supplementary Tables S3–S6**.

#### 3.4.2 Identification of somatic SNVs and INDELs

Somatic mutations have great value in explaining tumorigenesis and progression. We identified a total of 15892 somatic SNVs and 364 somatic INDELs, which were mainly clustered in the intronic and intergenic regions. Detailed information on the somatic SNVs and INDELs is presented in **Supplementary Tables S7–S8**.

#### 3.4.3 Identification of somatic SVs and CNVs

**Figure 9:**
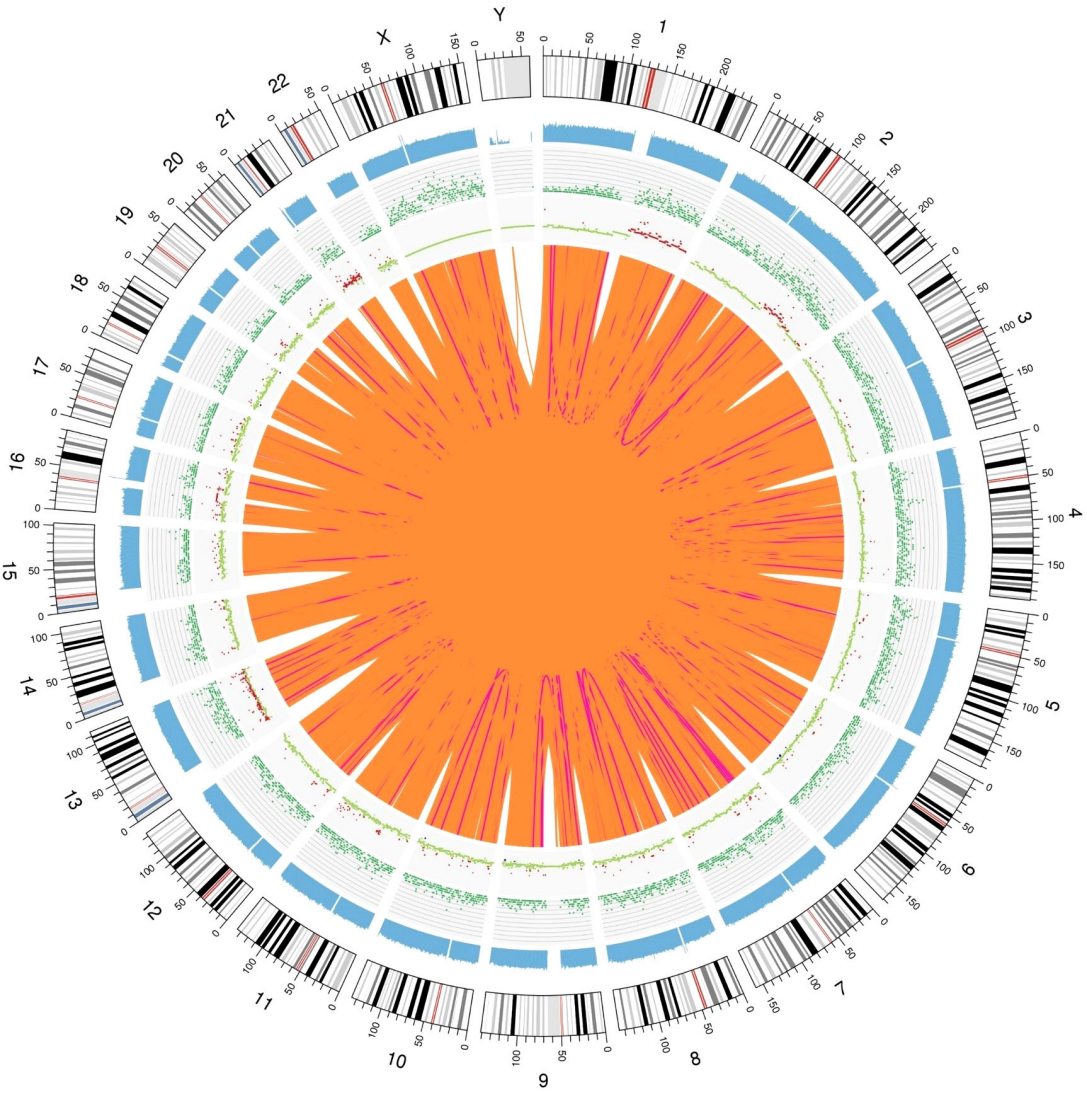
Genomic variation Circos plot. The structure of the three layers that run from the outside to the inside represents the sequence coverage map, SNP and INDEL density, and CNV results, respectively. Red indicates an increase in the copy number, blue indicates a decrease in the copy number, and green indicates a normal copy number.

In the detection of somatic SVs in the tumor specimens, we identified 238 duplicates, 247 deletions, 4 inversions, 24498 interchromosomal translocations and 2930 intrachromosomal translocations. A total of 572 somatic CNVs, including 548 gains and 24 losses, were identified. We show the Circos plot of somatic variations in **Figure 9**.

#### 3.4.4 Identification of Predisposing and Driver Mutating Genes

Mutations in cancer predisposition genes (CPGs) significantly increase the risk of cancer in individuals. We identified 24 predisposing genes, including HIP1, ELF1, and ZFHX3, among others, as shown in **Supplementary Table S9**. Identification of driver mutations and driver mutation genes that give tumors a selective growth advantage is important for understanding cancer pathogenesis. We identified 10 driver mutation genes, namely, LEPR, ACVR1, STAT3, TET2, IDH1, CHD7, STAT3, FAS and TP53, as shown in **Supplementary Table S10**.

#### 3.4.5 Analysis of tumor purity, ploidy and targeted drug prediction

The tumor purity was 0.4, the tumor ploidy was 3.39, and the cancer DNA fraction was 53%. We identified ten mutation sites associated with targeted drugs, and the detailed information is presented in **Table 2**. There were one drug (adenosine triphosphate) for ACVR1, five drugs (NADH, ethanol, fomepizole, cyclohexanol, glycerol) for ADH1B, seven drugs (enflurane, isoflurane, methoxyflurane, halothane, desflurane, sevoflurane and calcium) for ATP2C1, one drug (vorinostat) for HDAC8, six drugs (isocitric acid, FLT3 inhibitors, MEK inhibitors, JAK2 inhibitors, IDH1 inhibitors and VEGF antibodies/inhibitors) for IDH1, one drug (simvastatin) for LEPR, one drug (cyclosporine) for PPP3R2, one drug (bortezomib) for PSMB5, five drugs (bromodomain inhibitors, DOT1L inhibitors, other targeted epigenetic therapies, spliceosome-targeting therapies and Ras-targeting therapies) for TET2 and five drugs (bromodomain inhibitors, DOT1L inhibitors, other targeted epigenetic therapies, spliceosome-targeting therapies, and Ras-targeting therapies) for TP53.

**Table 2:** Prediction of targeted drugs based on somatic mutations.

Gene symbol	Entriz id	Chr	Start position	End position	Variant Classification	AA Change	Drug name	Drug type	Disease	Source
ACVR1	90	2	158622540	158622540	Missense_Mutation	ACVR1:NM_001105:exon8:c.A959C:p.H320P	adenosine triphosphate	small molecule	Gastroenterology	DrugBank
ADH1B	125	4	100235180	100235180	Missense_Mutation	ADH1B:NM_000668:exon6:c.T626C:p.V209A	nadh	small molecule	Gastroenterology	DrugBank
ADH1B	125	4	100235180	100235180	Missense_Mutation	ADH1B:NM_000668:exon6:c.T626C:p.V209A	ethanol	small molecule	Gastroenterology	DrugBank
ADH1B	125	4	100235180	100235180	Missense_Mutation	ADH1B:NM_000668:exon6:c.T626C:p.V209A	fomepizole	small molecule	Gastroenterology	DrugBank
ADH1B	125	4	100235180	100235180	Missense_Mutation	ADH1B:NM_000668:exon6:c.T626C:p.V209A	cyclohexanol	small molecule	Gastroenterology	DrugBank
ADH1B	125	4	100235180	100235180	Missense_Mutation	ADH1B:NM_000668:exon6:c.T626C:p.V209A	glycerol	small molecule	Gastroenterology	DrugBank
ATP2C1	27032	3	130656272	130656272	Missense_Mutation	ATP2C1:NM_001199182:exon4:c.G310C:p.A104P	enflurane	small molecule	Gastroenterology	DrugBank
ATP2C1	27032	3	130656272	130656272	Missense_Mutation	ATP2C1:NM_001199182:exon4:c.G310C:p.A104P	isoflurane	small molecule	Gastroenterology	DrugBank
ATP2C1	27032	3	130656272	130656272	Missense_Mutation	ATP2C1:NM_001199182:exon4:c.G310C:p.A104P	methoxyflurane	small molecule	Gastroenterology	DrugBank
ATP2C1	27032	3	130656272	130656272	Missense_Mutation	ATP2C1:NM_001199182:exon4:c.G310C:p.A104P	halothane	small molecule	Gastroenterology	DrugBank
ATP2C1	27032	3	130656272	130656272	Missense_Mutation	ATP2C1:NM_001199182:exon4:c.G310C:p.A104P	desflurane	small molecule	Gastroenterology	DrugBank
ATP2C1	27032	3	130656272	130656272	Missense_Mutation	ATP2C1:NM_001199182:exon4:c.G310C:p.A104P	sevoflurane	small molecule	Gastroenterology	DrugBank
ATP2C1	27032	3	130656272	130656272	Missense_Mutation	ATP2C1:NM_001199182:exon4:c.G310C:p.A104P	calcium	small molecule	Gastroenterology	DrugBank
HDAC8	55869	X	71710781	71710781	Missense_Mutation	HDAC8:NM_001166418:exon4:c.C353G:p.P118R	vorinostat	small molecule	Gastroenterology	DrugBank
IDH1	3417	2	209113113	209113113	Missense_Mutation	IDH1:NM_001282386:exon4:c.C394T:p.R132C	isocitric acid	small molecule	Gastroenterology	DrugBank
IDH1	3417	2	209113113	209113113	Missense_Mutation	IDH1:NM_001282386:exon4:c.C394T:p.R132C	flt3 inhibitors	–	acute-myeloid-leukemia	MycancerGenome
IDH1	3417	2	209113113	209113113	Missense_Mutation	IDH1:NM_001282386:exon4:c.C394T:p.R132C	mek inhibitors	–	acute-myeloid-leukemia	MycancerGenome
IDH1	3417	2	209113113	209113113	Missense_Mutation	IDH1:NM_001282386:exon4:c.C394T:p.R132C	jak2 inhibitors	–	acute-myeloid-leukemia	MycancerGenome
IDH1	3417	2	209113113	209113113	Missense_Mutation	IDH1:NM_001282386:exon4:c.C394T:p.R132C	idh1 inhibitors	–	glioma	MycancerGenome
IDH1	3417	2	209113113	209113113	Missense_Mutation	IDH1:NM_001282386:exon4:c.C394T:p.R132C	vegf antibodies/inhibitors	–	glioma	MycancerGenome
LEPR	3953	1	66096026	66096026	Missense_Mutation	LEPR: NM_001198687:exon19:c.C2815T:p.L939F	simvastatin	Efficacy	Hyperlipidemias	PharmGKB
LEPR	3953	1	66096026	66096026	Missense_Mutation	LEPR: NM_001198687:exon19:c.C2815T:p.L939F	simvastatin	Efficacy	Coronary Disease	PharmGKB
LEPR	3953	1	66096026	66096026	Missense_Mutation	LEPR: NM_001198687:exon19:c.C2815T:p.L939F	simvastatin	Efficacy	Hyperlipidemias	PharmGKB
PPP3R2	5535	9	104357190	104357190	Missense_Mutation	PPP3R2:NM_147180:exon1:c.C23G:p.A8G	cyclosporine	biotech	Gastroenterology	DrugBank
PSMB5	5693	14	23502876	23502876	Missense_Mutation	PSMB5:NM_001144932:exon2:c.A206G:p.H69R	bortezomib	small molecule	Gastroenterology	DrugBank
TET2	54790	4	106157174	106157174	Missense_Mutation	TET2:NM_001127208:exon3:c.A2075G:p.E692G	bromodomain inhibitors	–	myelodysplastic-syndromes	MycancerGenome
TET2	54790	4	106157174	106157174	Missense_Mutation	TET2:NM_001127208:exon3:c.A2075G:p.E692G	dot1l inhibitors	–	myelodysplastic-syndromes	MycancerGenome
TET2	54790	4	106157174	106157174	Missense_Mutation	TET2:NM_001127208:exon3:c.A2075G:p.E692G	or other targeted epigenetic therapies	–	myelodysplastic-syndromes	MycancerGenome
TET2	54790	4	106157174	106157174	Missense_Mutation	TET2:NM_001127208:exon3:c.A2075G:p.E692G	spliceosome-targeting therapies	–	myelodysplastic-syndromes	MycancerGenome
TET2	54790	4	106157174	106157174	Missense_Mutation	TET2:NM_001127208:exon3:c.A2075G:p.E692G	ras-targeting therapies	–	myelodysplastic-syndromes	MycancerGenome
TP53	7157	17	7578394	7578394	Missense_Mutation	TP53:NM_001126115:exon1:c.A140G:p.H47R	bromodomain inhibitors	–	myelodysplastic-syndromes	MycancerGenome
TP53	7157	17	7578394	7578394	Missense_Mutation	TP53:NM_001126115:exon1:c.A140G:p.H47R	dot1l inhibitors	–	myelodysplastic-syndromes	MycancerGenome
TP53	7157	17	7578394	7578394	Missense_Mutation	TP53:NM_001126115:exon1:c.A140G:p.H47R	or other targeted epigenetic therapies	–	myelodysplastic-syndromes	MycancerGenome
TP53	7157	17	7578394	7578394	Missense_Mutation	TP53:NM_001126115:exon1:c.A140G:p.H47R	spliceosome-targeting therapies	–	myelodysplastic-syndromes	MycancerGenome
TP53	7157	17	7578394	7578394	Missense_Mutation	TP53:NM_001126115:exon1:c.A140G:p.H47R	ras-targeting therapies	–	myelodysplastic-syndromes	MycancerGenome

Chr, chromosome; AA, amino acid; N/A, not applicable.

#### 3.4.6 Sanger sequencing

**Figure 10:**
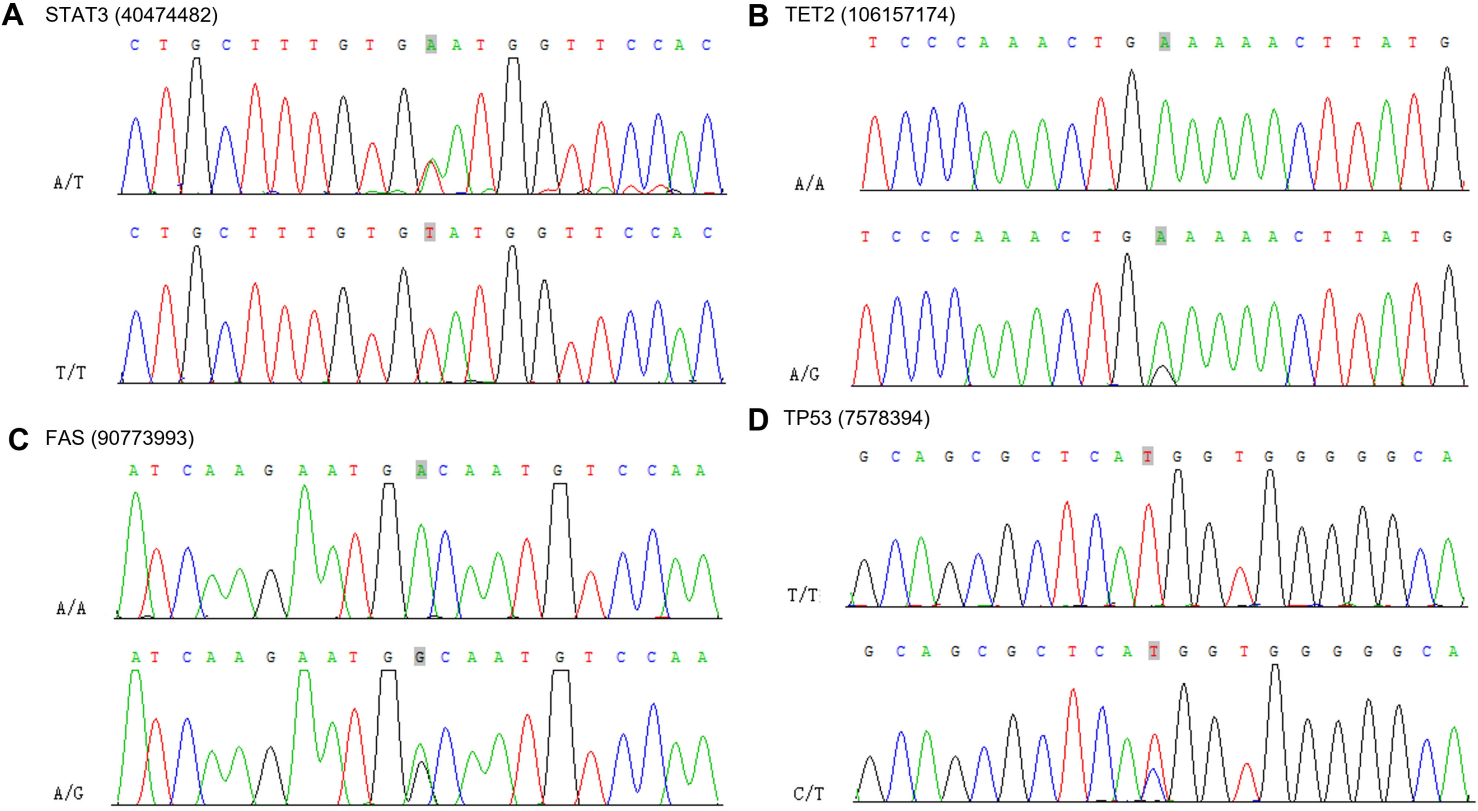
Sanger sequencing electropherograms of the STAT3 mutant at position chr17: 40474482 A>T **(A)**, TET2 mutant at position chr4: 106157174 G>A **(B)**, FAS mutant at position chr10: 90773993 G>A **(C)** and TP53 mutant at position chr17: 7578394 C>T **(D)**.

To confirm the presence of mutations in the susceptibility and driver genes, we performed Sanger sequencing. We confirmed the mutation of STAT3 at chr17: 40474482 A>T, TET2 at chr4: 106157174 G>A, FAS chr10: 90773993 G>A and TP53 at chr17: 7578394 C>T (**Figure 10**).

#### 3.4.7 Gene mutation analysis across cancers

**Figure 11:**
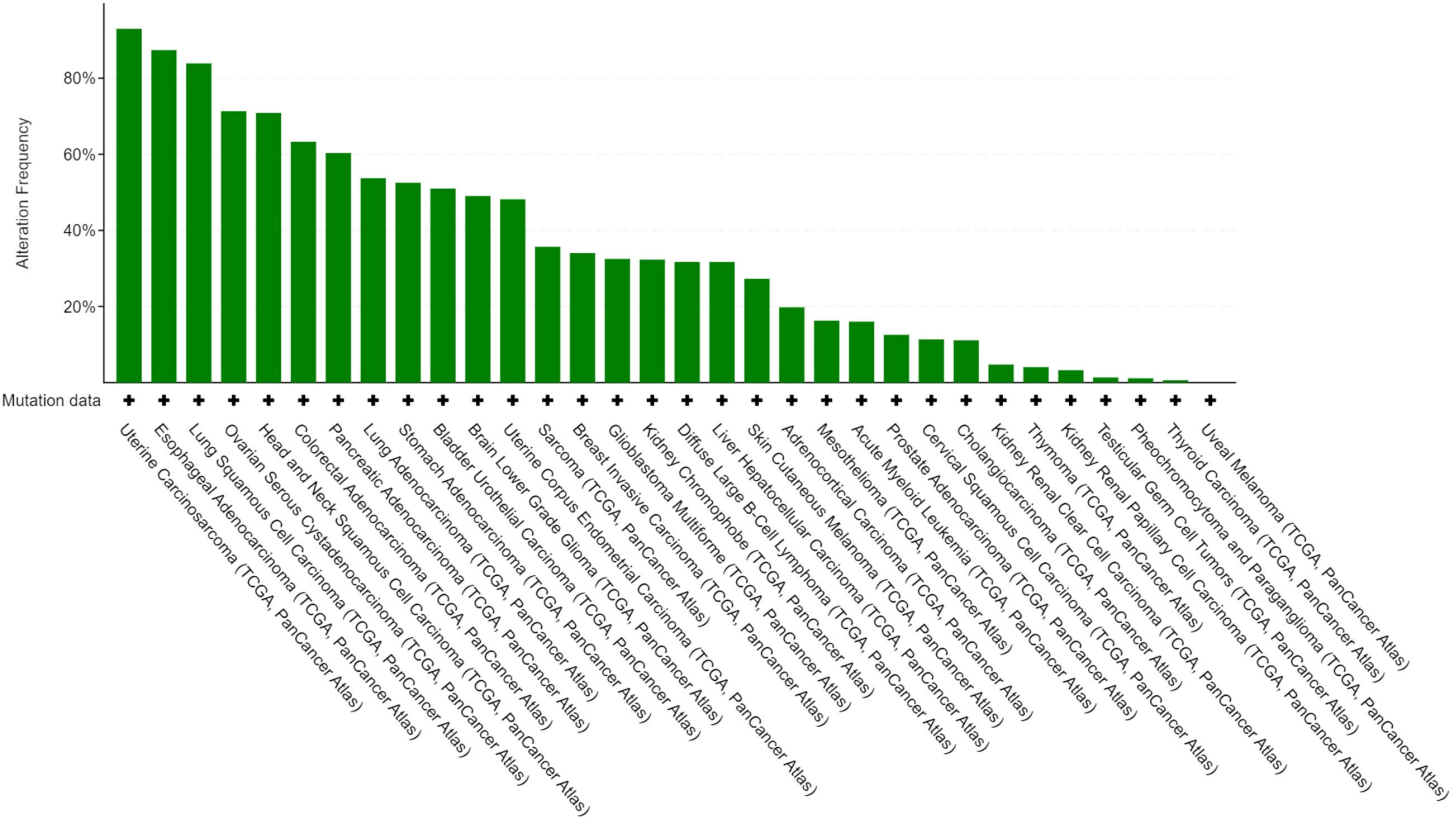
Alteration frequency of STAT3, TET2, FAS and TP53 in different studies.

**Figure 12:**
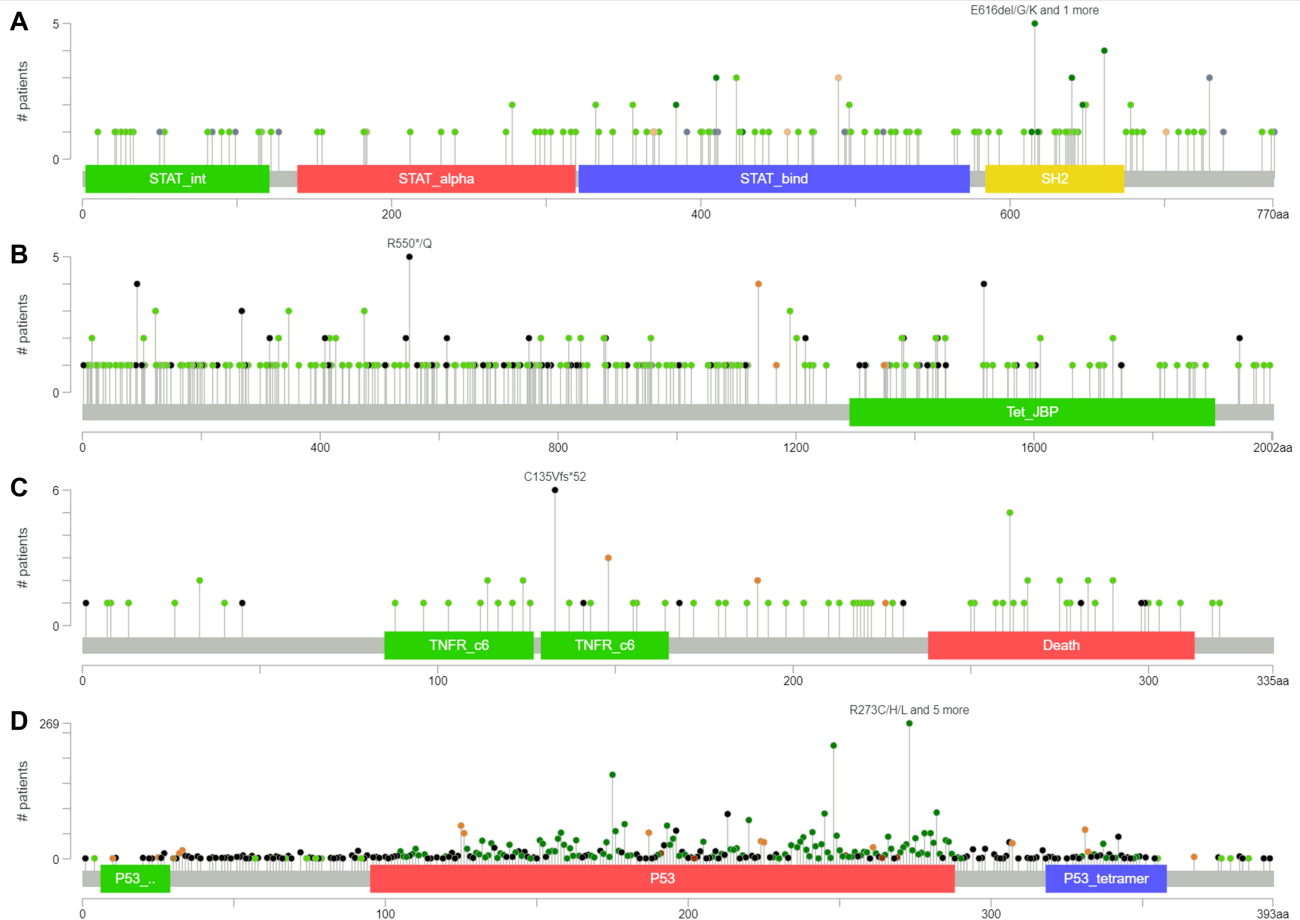
Lollipop plots of gene mutations. **(A)** STAT3. **(B)** TET2. **(C)** FAS. **(D)** TP53.

**Figure 13:**
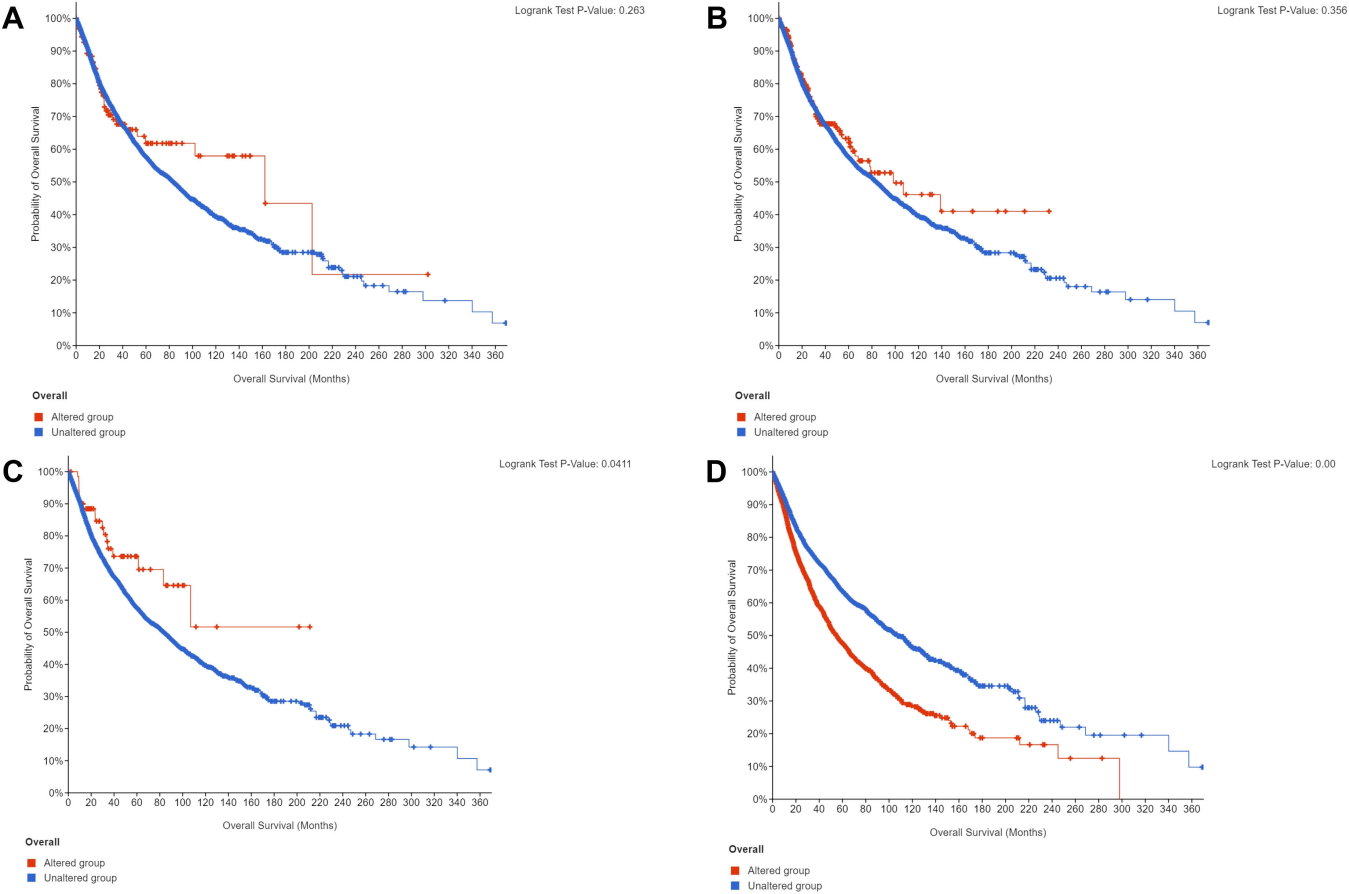
Overall survival curves according to gene mutation status. **(A)** STAT3. **(B)** TET2. **(C)** FAS. **(D)** TP53.

We performed gene mutation analysis of STAT3, TET2, FAS and TP53 in pancancer datasets, including 10967 samples from 32 studies, through the online database cBioPortal. The oncoprints showing the mutation types and cancer type summaries are presented in **Figure 11**. The mutual exclusivity analysis shown in **Table 3** revealed that mutations in STAT3, TET2 and TP53 tended to cooccur between any two genes (*p*<0.001). The lollipop figures in **Figure 12** present the mutation positions of the four genes. **Figure 13** shows that mutations in FAS (*p*=0.0411) and TP53 (*p*<0.001) were associated with OS.

**Table 3:** Mutual exclusivity.

A	B	Neither	A Not B	B Not A	Both	Log2 Odds Ratio	p-Value	q-Value	Tendency
TET2	FAS	10144	223	55	21	>3	<0.001	<0.001	Co-occurrence
STAT3	TET2	10084	115	223	21	>3	<0.001	<0.001	Co-occurrence
STAT3	FAS	10238	129	69	7	>3	<0.001	<0.001	Co-occurrence
FAS	TP53	6553	51	3814	25	-0.248	0.551	0.827	Mutual exclusivity
TET2	TP53	6451	153	3748	91	0.034	0.893	1	Co-occurrence
STAT3	TP53	6518	86	3789	50	0	1	1	Co-occurrence

A positive value of log2 odds ratio suggests that alterations in these genes co-occur in the same samples, while a negative value suggests that alterations in these genes are mutually exclusive and occur in different samples.

#### 3.4.8 Structure of the protein–protein interaction network and functional enrichment

**Figure 14:**
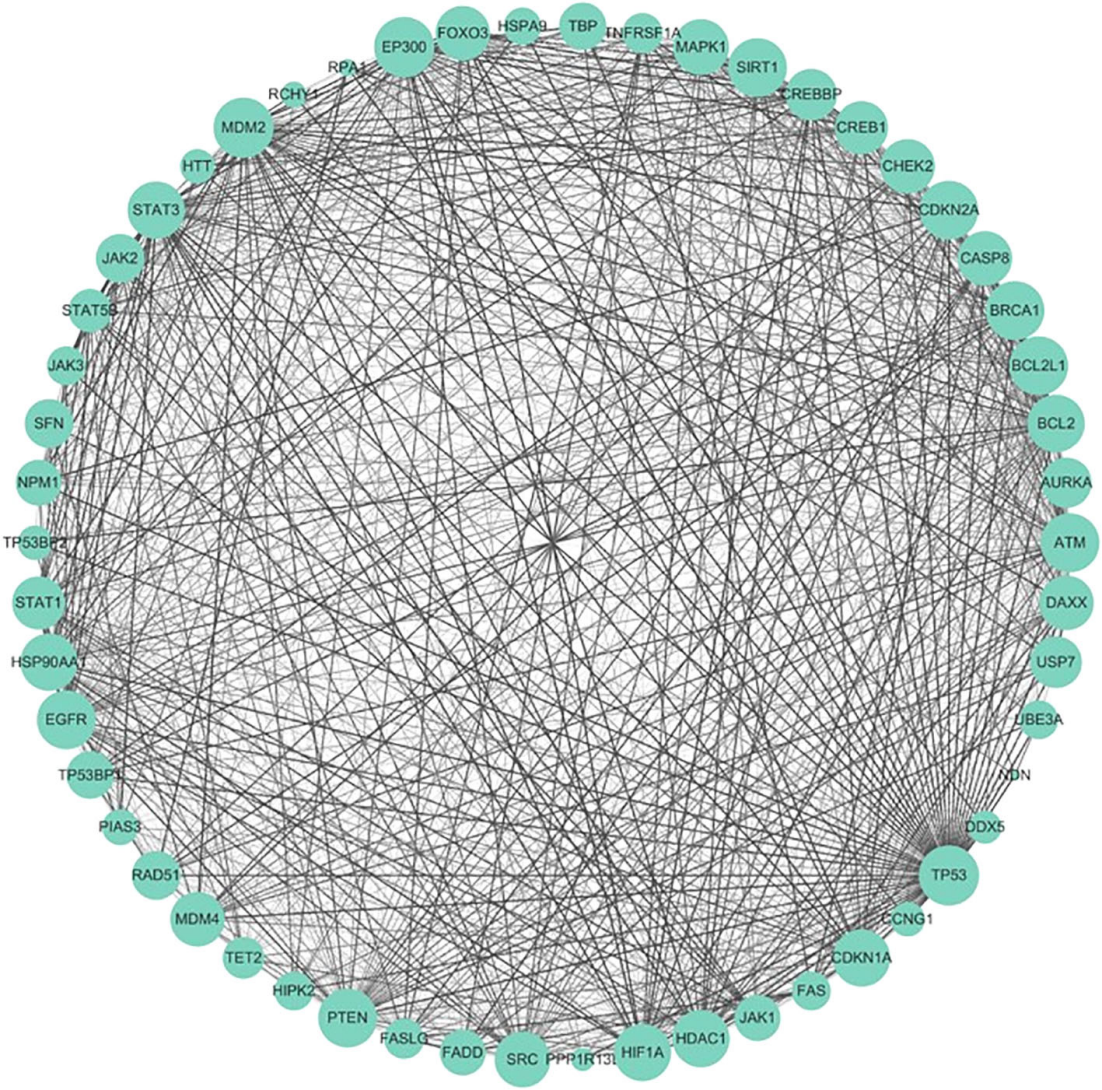
Protein–protein interaction network (PPI). The size of the nodes represents the degree of the nodes, and the thickness and color of the edge represents the score.

**Figure 15:**
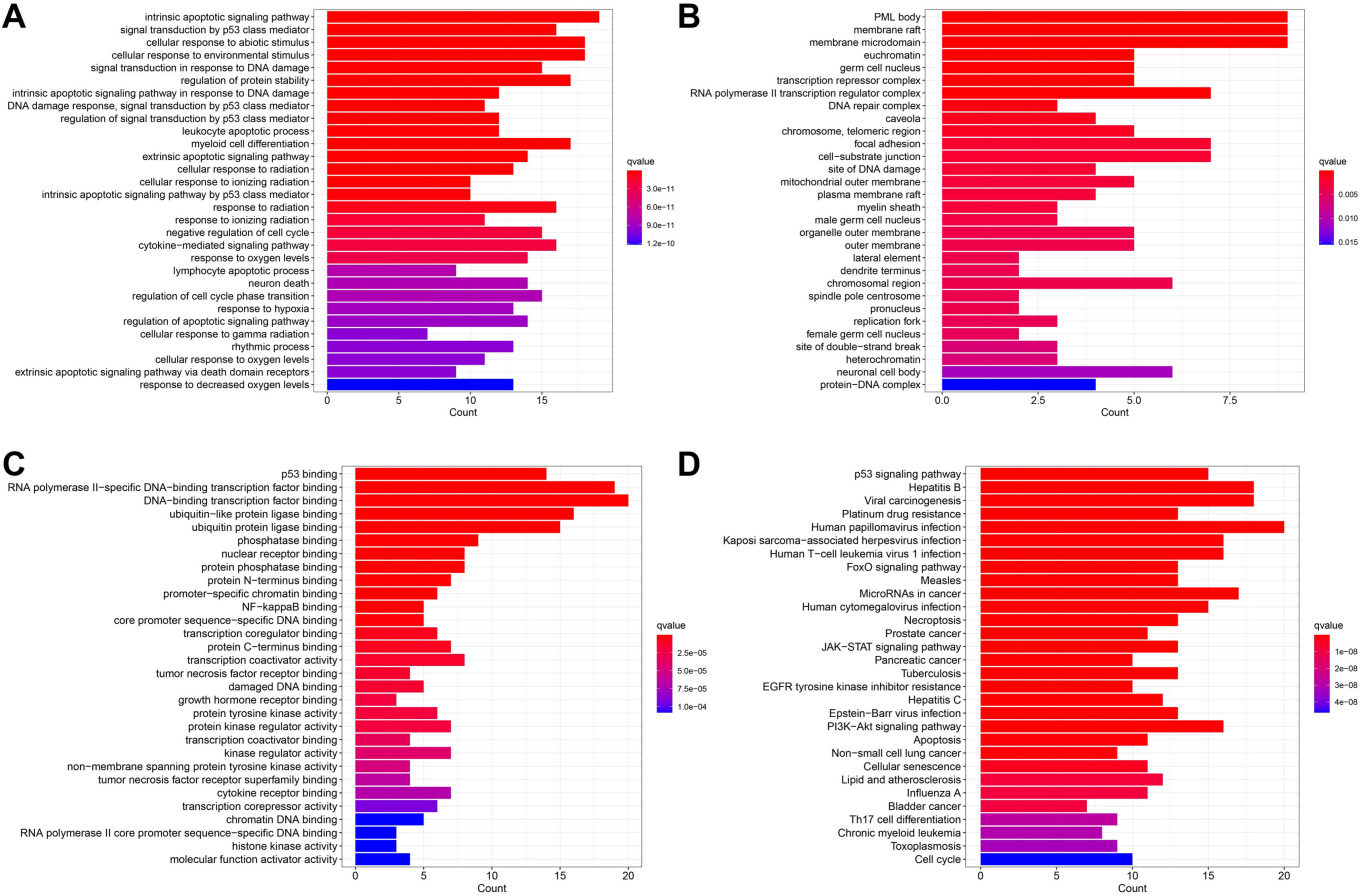
Enrichment analysis results. **(A)** The BP category of GO analysis. **(B)** The CC category of GO analysis. **(C)** The MF category of GO analysis. **(D)** KEGG pathway enrichment analysis.

The PPI network (**Figure 14**) contained 54 nodes and 119 edges. The GO analysis results revealed that the node genes were markedly enriched in the intrinsic apoptotic signaling pathway and extracellular matrix organization in the biological process category, the PML body and membrane raft in the cellular component category, and p53 binding in the molecular function category (**Figure 15**). According to the KEGG analysis, the genes were significantly enriched in the p53 signaling pathway (**Figure 15**).

## 4 Discussion

Adrenal tumors diagnosed as ENKTL are very rare, and patients tend to have a poor prognosis. In our study, we present a patient whose primary tumor site was nasal with secondary adrenal and systemic metastases. Despite being Ann Arbor stage IV and EBV positive as well as having a very high-risk NRI (age >60 years, stage IV, primary tumor invasion), after surgery, systemic therapy and immunotherapy, the patient was in good general condition with no signs of recurrence during 78 months of regular follow-up. To the authors’ knowledge, this is the longest surviving ENKTL patient with adrenal gland involvement according to a review of the literature on adrenal ENKTL patients. To determine the clinicopathological characteristics and prognosis of adrenal ENKTL patients, we enrolled 1771 ACC patients and 31 adrenal ENKTL patients after searching the PubMed, Medline, Cochrane Library, Web of Science, Embase, Scopus and SEER databases. The sex (p<0.001), race (p<0.001), laterality (p<0.001), tumor size (p<0.001), surgery (p<0.001) and chemotherapy (p<0.001) characteristics of the ENKTL patients were different from those of the ACC patients. According to the prognosis analyses, compared with ACC patients, ENKTL patients had a significantly poorer prognosis (p<0.001). The prognostic analyses of OS in patients with ENKTL showed that chemotherapy was an independent factor influencing the prognosis of ENKTL patients, which suggested that routine chemotherapy for ENKTL patients can improve prognosis and prolong patient survival. In the immunohistochemistry for detecting components of the tumor microenvironment, the infiltration of vascular endothelial cells and TAMs was detected, indicating the participation of vascular endothelial cells and TAMs in the pathogenesis of ENKTL. In the complex tumor microenvironment, inflammatory cells exert multifaceted and context-dependent effects on cancer progression, with TAMs representing a critical cellular component that exhibits distinct phenotypic plasticity and pro-tumorigenic functional properties across diverse malignancies (22–25). In the immunohistochemical analysis of CD68+ TAMs in 70 ENKTL patients Wang et al. performed demonstrated that high TAMs infiltration (>60/high-power field) was significantly associated with adverse clinical features, including B symptoms, elevated LDH levels, high Ki-67 expression, and lower complete response rates (46.7% vs 77.5%, *p*=0.008). Subsequent survival analysis revealed that patients in the high-TAM group had significantly worse 5-year overall survival (OS: 43.3% vs 79.5%) and progression-free survival (PFS: 30.0% vs 60.9%) compared to the low-TAM group (all *p*<0.01). Multivariate Cox regression analysis further identified CD68+ TAMs as an independent poor prognostic factor (OS: HR=3.195; PFS: HR=2.890) (26). TAMs may be involved in ENKTL progression by promoting tumor proliferation, inhibiting apoptosis, and mediating chemoresistance, suggesting that targeting TAMs may be a novel strategy for ENKTL patients.

We conducted WGS to detect genetic alterations in the patient, and validation by Sanger sequencing confirmed that STAT3, TET2, FAS and TP53 were the 4 main mutant driver genes. TP53 encodes the protein P53, which regulates the expression of the corresponding target genes in response to various cellular stresses, inducing the regulation of DNA repair, apoptosis, senescence, and metabolism (27). More than 50% of cancer patients carry TP53 mutations (28), and the most common type of mutation in TP53 is a missense mutation, which encodes a mutant P53 protein that not only inhibits the oncogenic effects of wild-type P53 but also exhibits new oncogenic functions, such as the promotion of cell proliferation, evasion of apoptosis, metabolic changes, and migration (27). TET2 is an epigenetic regulator that mediates DNA demethylation, promotes gene transcription and is crucial for host hematopoiesis and immunity (29). TET2 is frequently mutated in the hematopoietic system, eventually leading to the development of a series of blood malignancies (30). STAT3 is usually activated by IL-6 family cytokines via JAK and is involved in multiple cellular pathways, which in turn affects the proliferation, progression and immune dysfunction of cancer cells. STAT3 is usually considered an oncogene but may act as a tumor suppressor under specific circumstances. The aberrant activation of STAT3 occurs in approximately 70% of tumors. Inhibition of STAT3 in cancer not only decreases cell survival but can also reactivate the anticancer immune response, providing dual benefits (31). Accumulating evidence shows that STAT3 mutations participate in cancer pathogenesis, including in ENKTL. STAT3 mutations were first identified in hyper-IgE syndrome (32), and STAT3 mutations in solid tumors were detected in the research of Pilati et al. on inflammatory hepatocellular adenomas (33). The JAK-STAT signaling pathway is an evolutionarily conserved mechanism that mediates cellular responses to cytokines, interferons, and growth factors, regulating key processes such as immune function, inflammation, cell proliferation, and differentiation. Dysregulation of this pathway is strongly linked to autoimmune disorders fox example rheumatoid arthritis, atopic dermatitis and cancers including myeloproliferative neoplasms, prostate and breast cancer (34, 35). In next-generation sequencing of 34 ENKL samples, the JAK/STAT pathway was altered in 55.9% of cases, predominantly through STAT3 mutations in the SH2 domain that likely induce constitutive pathway activation. The genetic alterations may be further amplified by EBV infection, which enhances STAT3 signaling and reinforces the pathway oncogenic role in ENKTL (36). The key role of JAK/STAT signaling pathway in the pathogenesis of ENKTL was also been suggested by the previous researches (37, 38). Targeting JAK-STAT with inhibitors for example ruxolitinib shows therapeutic potential, particularly in myeloproliferative neoplasms and leukemias, though challenges remain in understanding its full regulatory mechanisms and optimizing clinical applications (39). The binding of FAS, a transmembrane receptor, to its natural ligand (FasL) plays a pivotal role in immune homeostasis and surveillance. Mutations in FAS (40), mainly at the site of the fine death receptor structural domain involved in the recruitment of the connexin FAS-associated protein to the death structural domain, have been found to be associated with the disruption of self-tolerance in patients with autoimmune lymphoproliferative syndrome. FAS mutations have also been found in lymphomas, suggesting that FAS may be a tumor suppressor gene. Death receptor structural domain site mutations promote tumor progression by rendering tumor cells resistant to apoptotic responses but may also promote tumor progression by promoting tumorigenesis and inflammatory or autoimmune mechanisms (41). Takakuwa et al. found that approximately 50% of ENKTL patients harbored FAS gene deletions or mutations, which impaired the formation of the death-inducing signaling complex, leading to inactivation of the FAS signaling pathway and enabling tumor cells to evade apoptosis (42, 43). Moreover, the EBV-encoded LMP1 protein activates both NF-κB and PI3K/Akt pathways, leading to upregulation of anti-apoptotic proteins (such as c-FLIP and Bcl-2), which indirectly suppresses FAS-mediated apoptosis. The combined effects of FAS functional defects and EBV oncoproteins in promoting immune evasion may contribute to disease progression in ENKTL (44, 45). FAS mutations result in defective apoptotic signaling in ENKTL cells, serving as a critical mechanism for immune evasion and tumor cell survival. Targeting the pathway through combinatorial approaches, such as synergizing immune checkpoint inhibitors with apoptosis-inducing therapeutics, represents a promising treatment strategy for ENKTL patients. In ENKTL patients, mutations in TP53, STAT3 and TET2 are frequent, although the proportion of these mutations is heterogeneous across studies. TP53 mutations are associated with increased mortality in ENKTL patients (27) and is the most frequent mutation in relapsed patients (46). Recurrent mutations in genes in the JAK-STAT pathway, including STAT3, play an important role in ENKTL survival and proliferation and the development of precise therapeutic approaches for JAK/STAT inhibition (47). Moreover, STAT3 mutation has been investigated in EBV-positive inflammatory follicular dendritic cell sarcoma and EBV-positive HIV-associated diffuse large B-cell lymphoma (48, 49), and we believe that chronic active EBV infection may play an important role in the role of STAT3 mutation in activating tumor growth. As an epigenetic biomarker of ENKTL, TET2 mutation is significantly associated with the loss of protein expression and is correlated with shorter overall survival, suggesting that TET2 may be involved in the cancer-driving process of ENKTL (50).

For patients with stage III/IV or relapsed/refractory disease, asparaginase‐based chemotherapy is the primary treatment (51). Regimens containing L-asparaginase, including SMILE and AspaMetDex (L-asparaginase, methotrexate, and dexamethasone), exhibited similar efficiency, while patients showed better tolerance to the AspaMetDex regimen (52). The P-GEMOX and DDGP (dexamethasone, cisplatin, gemcitabine, and pegaspargase) regimens involving the substitution of L-asparaginase, such as pegylated asparaginase, have shown promising results. Compared with SMILE, P-GEMOX and DDGP resulted in a better treatment response and survival benefit and exhibited reduced toxicity (53). Moreover, the GDP regimen (gemcitabine, dexamethasone, and cisplatin) had equally superior efficacy and reduced toxicity, including a lower incidence of myelosuppression, prolonged activated partial thromboplastin time, pancreatitis, and anaphylactic reaction, in comparison to L-asparaginase treatment (54). The therapeutic efficacy and good tolerance of monotherapy with PD-1/PD-L1 antibodies in ENKTL patients who did not respond to asparaginase-based treatment have been demonstrated in early-phase trials and retrospective studies (55, 56). The efficacy of PD-1 antibody (sintilimab) combined with P-GEMOX was highly promising, achieving an 85% complete response rate and 100% overall response rate in the research of Tian et al. (57), consistent with previous reports demonstrating an 88.9% objective response rate (58), further confirming the robust antitumor activity of this regimen in advanced ENKTL with durable survival benefits and a manageable safety profile. A retrospective study by Wang X et al. also revealed that immunochemotherapy with an anti-PD-1 antibody was an independent prognostic factor for longer PFS in ENKTL patients (*p*<0.001) (59). What’s more, PD-L1 monoclonal antibodies sugemalimab demonstrate promising efficacy in relapsed/refractory ENKTL, with a 35.9% complete response rate and durable survival benefits, while maintaining a manageable safety profile compared to PD-1 inhibitors including sintilimab, pembrolizumab, nivolumab and tislelizumab (60). LMP, which is expressed in the tumor cells of EBV-associated malignancies, is a natural candidate target for cytotoxic T lymphocytes (61), and patients who received LMP-specific cytotoxic T cells achieved better PFS and OS and less toxicity (62, 63). Some new therapeutic targets explored by NGS have also shown excellent potential for the treatment of ENKTL. Daratumumab, a monoclonal antibody against CD38, and brentuximab vedotin, an antibody targeting CD30 conjugated to the cytotoxic agent monomethyl auristatin E, both showed promising activity in ENKTL patients (62, 64, 65). The efficacy of ruxolitinib, a JAK3-STAT pathway inhibitor, in peripheral T-cell lymphoma patients has been confirmed (66), while a clinical trial evaluating ruxolitinib in ENKTL patients is ongoing (NCT02974647) (6). Our WGS analysis confirmed that the STAT3 mutation may be a potential therapeutic target for this patient. The benefit of hematopoietic stem cell transplantation in patients with advanced-stage or relapsed disease has been demonstrated in several small, retrospective studies, but prospective, randomized clinical trials are needed (67–69). Moreover, several potential targets, such as EZH2, BCOR, MLL2, NF-kappaB, NOTCH, Bcl-xL, TET1/TET2, EP200, ASXL3, CREBBP and ARID1A, have also been investigated (69–71).

There were some limitations in our study. Given the rarity of ENKTL with adrenal involvement, the statistical power of our WGS findings may be constrained, sequencing studies with large samples may be helpful for systematically elucidating the genetic alterations of adrenal ENKTL to enhance the generalizability and reproducibility of our conclusions. What’s more, the cells and animal experiments are also required to perform to validate the role of genetic alterations in the ENKTL involved adrenal mechanisms. In the analyses of prognostic and clinicopathological factors, the results may have been affected by confounding factors due to the limited sample size, which restricts the ability to match ENKTL patients and ACC patients by propensity matching scores. Moreover, the limited information available for patients in the SEER database hindered comprehensive analysis of significant factors and the efficacy of immunotherapy and therapeutic targets. Studies with larger samples may be more helpful in comparing survival between ENKTL patients and ACC patients and in exploring risk factors affecting survival in ENKTL patients.

## 5 Conclusion

In conclusion, adrenal ENKTL is very rare, and this study described a patient with primary nasal tumors and secondary adrenal and multiple systemic metastases. Four mutation driver genes, STAT3, TET2, FAS and TP53, were identified using WES and Sanger sequencing, which may contribute to targeted therapy for this disease. According to our prognostic analysis, patients with adrenal ENKTL presented a significantly worse prognosis than patients with ACC, and administering chemotherapy improved the prognosis of adrenal ENKTL patients.

## Data Availability

To protect the privacy of the sampled individual, the SNP data presented in this article are not readily available. Requests to access the dataset should be directed to the corresponding author Bo Fan.
