# Supplementary material for: Functional screening of somatic mutant events in extranodal natural killer/T-cell lymphoma with adrenal involvement
Source: Front Immunol. 2025 May 13;16:1566794. doi: 10.3389/fimmu.2025.1566794 (PMC12106576; doi:10.3389/fimmu.2025.1566794)
Supplement: Supplementary file 1 [file DataSheet1.docx]

**Supplementary materials**

**Table S1.** Antibodies used in the immunohistochemistry. Ig, Immunoglobulin; CD, cluster of differentiation.

| **Antibody** | **Species** | **Antibody number** | **Source** | **Dilution** |  |
| --- | --- | --- | --- | --- | --- |
| IgG | Rabbit | ab200699 | Abcam, Cambridge, UK | 1:1000 |  |
| IgG4 | Mouse | 66408-1-Ig | Protein Tech Group, Chicago, USA | 1:5000 |  |
| IgGκ | Rabbit | 14678-1-AP | Protein Tech Group, Chicago, USA | 1:100 |  |
| lgGλ | Rabbit | 20758-1-AP | Protein Tech Group, Chicago, USA | 1:200 |  |
| CD3 | Rabbit | ab16669 | Abcam, Cambridge, UK | 1:100 |  |
| CD43 | Rabbit | 13959-1-AP | Protein Tech Group, Chicago, USA | 1:100 |  |
| CD56 | Mouse | MA1-06801 | ThermoFisher Scientific, Massachusetts, USA | 1:200 |  |
| TIA-1 | Mouse | MA5-26474 | ThermoFisher Scientific, Massachusetts, USA | 1:200 |  |
| Granzyme B | Rabbit | ab255598 | Abcam, Cambridge, UK | 1:3000 |  |
| CD2 | Rabbit | ab227698 | Abcam, Cambridge, UK | 1:100 |  |
| CD4 | Rabbit | ab183685 | Abcam, Cambridge, UK | 1:500 |  |
| CD5 | Rabbit | 17227-1-AP | Protein Tech Group, Chicago, USA | 1:800 |  |
| CD7 | Rabbit | MA5-43071 | ThermoFisher Scientific, Massachusetts, USA | 1:400 |  |
| CD8 | Mouse | 66868-1-Ig | Protein Tech Group, Chicago, USA | 1:6000 |  |
| CD20 | Rabbit | ab78237 | Abcam, Cambridge, UK | 1:100 |  |
| CD79α | Rabbit | ab79414 | Abcam, Cambridge, UK | 1:100 |  |
| Cytokeratin AE1/AE3 | Mouse | ab27988 | Abcam, Cambridge, UK | 1:20 |  |
| Ki67 | Rabbit | 27309-1-AP | Protein Tech Group, Chicago, USA | 1:6000 |  |
| PD-1 | | Mouse | ab52587 | Abcam, Cambridge, UK | 1:100 |
| PD-L1 | Rabbit | ab205921 | Abcam, Cambridge, UK | 2 μg ml−1 |  |
| CD31 | Rabbit | ab28364 | Abcam, Cambridge, UK | 1:50 |  |
| CD34 | Rabbit | ab110643 | Abcam, Cambridge, UK | 1:50 |  |
| CD163 | Rabbit | ab182422 | Abcam, Cambridge, UK | 1: 400 |  |
| CD68 | Mouse | ab955 | Abcam, Cambridge, UK | 1: 3000 |  |
| Vimentin | Rabbit | ab92547 | Abcam, Cambridge, UK | 1: 400 |  |
| SMA | Mouse | MA1-06110 | ThermoFisher Scientific, Massachusetts, USA | 1:200 |  |

**Table S2.** Detailed characteristic of adrenal extra-nodal natural killer/T -cell lymphoma. AASS: Ann Arbor staging system; LDH: lactic dehydrogen/ase; sIL-2R: soluble interleukin-2 receptor; GzB: granzyme B; Ki-67 LI: Ki-67 labeling index; EBER, EBV-encoded small RNAs; LVDP: L-asparagin/ase, etoposide, dexamethasone, and cisplatin; N/A: not available; MOGAD: methotrexate, oxaliplatin, gemcitabine, asparaginase, and dexamethasone; SMILE regimen: dexamethasone, methotrexate, ifosfamide, l-asparagin/ase and etoposide); CHOP: cyclophosphamide, hydroxydaunorubicin, oncovin, and prednisone; Or: orchiectomy; P-GEMOX: Peg-asparagin/ase, gemcitabine and oxaliplatin; CHOP-E: cyclophosphamide, doxorubicin, vincristine, prednisone and etoposide; P+: partial positive; VP-16: Synonyms; DXM: dexamethasone; hyperCVAD: hyperfractionated cyclophosphamide, vincristine, doxorubicin, dexamethasone; MA: methotrexate, cytarabine; DeVIC: carboplatin, etoposide, ifosfamide and dexamethasone; IMVP-16: ifosfamide, mesothrexate, etoposide; CHOP-L: cyclophosphamide, doxorubicin, vincristine, prednisone, and L-asparaginase; THP-COP: pirarubicin hydrochloride, vincristine sulfate, cyclophosphamide, predonine; N-BEPP: mitoxantrone hydrochloride , bleomycine hydrochloride, etoposide, procarbazine hydrochloride , predonine.

|  | Reference | Region | Age/ Sex | Laterality (max tumor diameter [mm]) | Primary tumor location | Subsequent sites involvement | Diagnostic measure | AASS | B symptoms | LDH (IU/L) | sIL-2R (U/mL) | Blood EBV-DNA (copies/µg) | Immunophenotype | | | | | | | | | | EBER-ISH | Ki-67 LI | Therapy | Follow-Up |
| --- | --- | --- | --- | --- | --- | --- | --- | --- | --- | --- | --- | --- | --- | --- | --- | --- | --- | --- | --- | --- | --- | --- | --- | --- | --- | --- |
|  |  |  |  |  |  |  |  |  |  |  |  |  | CD2 | CD3 | CD4 | CD5 | CD8 | CD20 | CD30 | CD56 | GzB | TIA-1 |  |  |  |  |
| 1 | Mnafe O 2024(1) | Morocco | 24/M | Bilateral (N/A) | testicular, adrenal | nasal | Biopsy | Ⅳ | Yes | Increased | N/A | N/A | N/A | + | - | - | - | N/A | - | + | N/A | N/A | N/A | 50% | MOGAD + autologous stem cell transplantation | Alive (12 mo) |
| 2 | Liu C 2023(2) | China | 57/F | Bilateral (N/A) | Skin, CNS, intestine, adrenal | Skin, CNS, intestine, adrenal | Biopsy | Ⅳ | No | 314 (Increased) | N/A | 5420 | + | + | N/A | - | - | + | - | + | + | + | + | 80% | P-GemOx + methotrexate +penpulimab+temozolomide+Selinexor+thalidomide+etoposide | Alive (10 mo) |
| 3 | Sekar A 2023(3) | India | 24/F | Bilateral (N/A) | nasal cavity and nasopharynx | kidneys, adrenals, liver, spleen, and small intestine | autopsy | Ⅳ | Yes | N/A | N/A | N/A | N/A | + | N/A | N/A | N/A | - | N/A | + | N/A | N/A | + | N/A | - | Dead (6 mo) |
| 4 | Zhou SL 2023 (1)(4) | China | 57/M | Bilateral (68) | Adrenal gland | No | CT -guided biopsy | Ⅰ | Yes | Increased | N/A | N/A | + | + | N/A | N/A | N/A | - | N/A | + | N/A | + | + | >80% | Chemotherapy | Alive (4 mo) |
| 5 | Zhou SL 2023 (2)(4) | China | 67/M | Bilateral (93) | Adrenal gland | No | CT -guided biopsy | Ⅰ | No | Normal | N/A | N/A | + | + | N/A | N/A | N/A | - | N/A | + | N/A | + | + | >80% | Chemotherapy | Dead (3 mo) |
| 6 | Zhou SL 2023 (3)(4) | China | 58/F | Unilateral (87) | Adrenal gland | No | Surgical resection | Ⅰ | No | Increased | N/A | N/A | + | + | N/A | N/A | N/A | - | N/A | + | N/A | + | + | >80% | Surgery | Alive (42 mo) |
| 7 | Zhou SL 2023 (4)(4) | China | 59/F | Bilateral (58) | Adrenal gland | No | Surgical resection | Ⅰ | Yes | Increased | N/A | N/A | + | + | N/A | N/A | N/A | - | N/A | + | N/A | + | + | >80% | Surgery + chemotheraoy | Dead (7 mo) |
| 8 | Zhou SL 2023 (5)(4) | China | 66/F | Bilateral (72) | Adrenal gland | No | CT -guided biopsy | Ⅰ | No | Increased | N/A | N/A | + | + | N/A | N/A | N/A | - | N/A | + | N/A | + | + | >80% | Chemotherapy | Dead (11 mo) |
| 9 | Ji XY 2023(5) | China | 55/M | Bilateral (L62, R N/A) | Adrenal gland | No | Surgical resection | Ⅰ | Yes | 231 | N/A | 2.24 × 10^4^ | N/A | + | N/A | N/A | N/A | - | - | + | + | + | + | 80% | P-GemOx, VP-16, Sintilimab | Dead (17 mo) |
| 10 | Wu W 2023(6) | China | 62/F | N/A | Upper aerodigestive tract | Paranasal sinuses, bones, bone marrow, adrenal gland | Pathological examination | Ⅳ | Yes | Elevated | N/A | 6.56 × 10^4^ | N/A | ε+ | N/A | - | N/A | - | NA | + | + | + | + | 55% | LVDP | Dead (25 mo) |
| 11 | Baba Y 2022(7) | Japan | 32/M | Left | A left adrenal  mass with ill-defined margins infiltrating the pancreas and kidney | No | CT -guided  biopsy of the left adrenal mass | Ⅳ | Yes | 898 | 4984 | N/A | N/A | ε+ | + | N/A | N/A | N/A | N/A | + | + | + | + | N/A | SMILE | Dead (4 mo) |
| 12 | Ichikawa S 2020(8) | Japan | 37/M | Bilateral (L138, R 101) | Adrenal gland | internal  acoustic meatuses and the fourth ventricle | CT-guided needle biopsy of left adrenal | Ⅳ | Yes | 394 | 4155 | 5.1 × 10^4^ | + | ε+ | - | - | - | - | - | + | + | + | + | 95% | SMILE, Sib/ PBSCT | Dead (10 mo) |
| 13 | Yu P 2020(9) | China | 48/M | Right (N/A) | Sacrum | right adrenal gland and retroperitoneal lymph nodes, left cervical lymph node | biopsy of the left cervical lymph node | Ⅳ | Yes | N/A | N/A | N/A | + | + | - | N/A | - | - | + | + | + | + | + | 70% | Sacral tumor resection, CHOP, MEOP, ESHAP, topical radiotherapy, | Alive (70mo) |
| 14 | Dong P 2019(10) | China | 40/F | Bilateral (L75, R 31) | Adrenal gland, soft tissue | No | Surgical resection of left adrenalectomy and  ipsilateral nephrectomy | Ⅰ | No | N/A | N/A | N/A | + | ε+ | - | - | - | - | + | + | + | N/A | + | 70% | left adrenalectomy and ipsilateral nephrectomy, GLIDE, HDT+ASCT | Survival (>1y) |
| 15 | Han Y 2019(11) | Korea | 46/M | Bilateral (L60, R 60) | Adrenal gland, pericardium | No | A percutaneous, transabdominal needle biopsy (PTNB) at the left adrenal gland | Ⅳ | Yes | 678 | N/A | N/A | N/A | + | N/A | N/A | N/A | N/A | N/A | + | N/A | + | + | N/A | CHOP | Dead (3 w) |
| 16 | Huang Y 2019 (1)(12) | China | 48/M | N/A | Testis | Adrenal gland,skin, lymph node | Surgical resection of orchiectomy | Ⅳ | No | N/A | N/A | N/A | N/A | ε+ | N/A | - | N/A | - | - | + | + | + | + | 90% | P-GEMOX | Dead (4 mo) |
| 17 | Huang Y 2019 (2)(12) | China | 67/M | N/A | Testis | Adrenal gland, lung | Surgical resection of orchiectomy | Ⅳ | No | N/A | N/A | N/A | N/A | ε+ | N/A | - | N/A | + | - | + | + | + | + | 90% | P-GEMOX | Alive (4 mo) |
| 18 | Huang Y 2019 (3)(12) | China | 41/M | N/A | Testis | Adrenal gland, bone marrow | Surgical resection of orchiectomy | Ⅳ | No | N/A | N/A | N/A | N/A | ε+ | N/A | - | N/A | - | P+ | + | + | + | + | 70% | P-GEMOX | Alive (52 mo) |
| 19 | Huang Y 2019 (4)(12) | China | 60/M | N/A | Testis | Adrenal gland, lymph node | Surgical resection of orchiectomy | Ⅳ | No | N/A | N/A | N/A | N/A | ε+ | N/A | - | N/A | - | N/A | + | - | + | + | 80% | CHOP-E | Dead (19 mo) |
| 20 | Huang J 2018(13) | China | 44/M | Bilateral | Adrenal gland invading  the top of renal and partial of pancreas | No | CT-guided needle biopsy of adrenal mass | Ⅳ | Yes | 1346 | N/A | 1.22×10^4^ | N/A | + | N/A | N/A | N/A | - | N/A | + | N/A | N/A | + | 70% | GemOx, VP-16+DXM | Dead (40 d) |
| 21 | Hu L 2017(14) | China | 28/M | Left (100) | Adrenal mass invading  the top of left renal and partial of retroperitoneal region | No | Surgical resection of adrenal mass | Ⅳ | No | N/A | N/A | N/A | + | dim | - | - | - | - | N/A | + | N/A | N/A | + | 40% | Surgical resection of adrenal mass, CHOP, Radiotherapy | Alive (26 mo) |
| 22 | Wang X 2017(15) | China | 58/M | N/A | Nasal, Penis | adrenal gland, liver, spleen and lymph nodes | Surgical resection of penile mass | Ⅳ | No | Normal | N/A | N/A | N/A | + | N/A | N/A | N/A | - | N/A | + | + | + | + | N/A | CHOP-L | Dead (4 mo) |
| 23 | Kabnurkar R 2016(16) | India | 41/M | Left (80) | Adrenal gland | No | CT-guided biopsy of the adrenal mass | Ⅰ | No | Increased | N/A | N/A | N/A | + | - | N/A | - | - | N/A | + | N/A | N/A | N/A | N/A | SMILE | Alive (3 mo) |
| 24 | Tian C 2015(17) | China | 52/M |  | many bones  of the whole body, adrenal gland | No | CT-guided biopsy of the  ilium lesion | Ⅳ | Yes | N/A | N/A | N/A | + | + | - | - | N/A | - | - | + | N/A | N/A | N/A | N/A | SMILE | Dead (1 m) |
| 25 | Liang DN 2012(18) | China | 44/M | Bilateral | Adrenal gland | Testis | Surgical resection (resected teste) | Ⅳ | Yes | N/A | N/A | N/A | N/A | ε+ | N/A | N/A | N/A | N/A | N/A | + | + | + | + | 80% | CHOP | Dead (6 m) |
| 26 | Poli M 2012(19) | France | 51/M | N/A | Eye frames, nasal cavity, paranasal sinus,  meningeal, renal, adrenal, and digestive | No | transnasal biopsies | Ⅳ | Yes | N/A | N/A | N/A | N/A | + | - | N/A | - | N/A | N/A | + | N/A | N/A | + | + | Systemic and intrathecal chemotherapy | Dead (a few weeks) |
| 27 | Tsukahara T 2012(20) | Japan | 79/F | Bilateral (L 85, R 90) | Bilateral adrenal gland, right kidney, inferior vena cava, left kidney and spleen | No | Autopsy | Ⅳ | Yes | 1038 | 1185 | N/A | N/A | + | - | - | - | - | - | - | - | + | + | 77% | - | Dead (33 d) |
| 28 | Ishihara S 2011(21) | Japan | 78/M | N/A | Adrenal gland, pericardium, lungs, pancreas | CNS | Laparoscopic biopsy of the adrenal gland | Ⅳ | Yes | N/A | 1850 | N/A | N/A | N/A | N/A | N/A | N/A | N/A | N/A | + | N/A | N/A | N/A | N/A | DeVIC + intravenous MTX pulse therapy, intrathecal administration of Ara-C, MTX and PSL | Alive (2 mo) |
| 29 | Kang SM 2011(22) | Korea | 29/M | Bilateral | Adrenal gland, soft tissue, lymph node | No | Surgical resection of cervical lymph node | Ⅳ | Yes | 1591 | N/A | N/A | N/A | N/A | N/A | N/A | N/A | - | + | + | N/A | N/A | N/A | + | IMVP-16 | Dead (59 d) |
| 30 | Nagireddy S 2011(23) | America | 70/M | Bilateral (L 81, R 55) | bilateral adrenal, retroperitoneal adenopathy, and in the pituitary gland | Meninges, left middle cranial fossa, paranasal sinus, breast, lungs, pleura, kidney, and prostate | Adrenal CT-guided needle biopsy | Ⅳ | Yes | N/A | N/A | N/A | N/A | + | - | N/A | - | N/A | N/A | + | N/A | N/A | + | N/A | Salvage chemotherapy | Dead (1 mo) |
| 31 | Dunning KK 2009(24) | America | 67/M |  | Adrenal gland, leptomeningeal | No | CT-guided  needle biopsy of right adrenal gland mass | Ⅳ | No | N/A | N/A | N/A | + | - | - | - | - | - | + | + | + | + | + | N/A | DXM | Dead (a few days) |
| 32 | Mozos A 2009(25) | Spain | 70/M | Bilateral (L 52, R 43) | Adrenal gland | No | Needle biopsy of adrenal gland mass | Ⅰ | Yes | Elevated | N/A | N/A | + | + | N/A | - | + | N/A | - | + | + | + | + | N/A | CHOP +  vincristine | Dead (3.7 mo) |
| 33 | Toba A 2008(26) | Japan | 76/F | Bilateral (L 90, R 50) | Adrenal gland, right ileocephalic region, left lower pelvis, nasal cavity, left lower jaw, upper vertical septum | No | nasal cavity mass biopsy | Ⅳ | Yes | 366 | 2730 | N/A | N/A | + | N/A | N/A | N/A | - | N/A | + | + | N/A | - | N/A | THP-COP, N-BEPP | Dead (2 mo) |
| 34 | Thompson MA 2007(27) | America | 35/M | Left (50) | Adrenal gland | No | Surgical resection of left open adrenalectomy | Ⅰ | Yes | 666 | N/A | N/A | dim | dim | N/A | - | N/A | dim | N/A | + | + | + | + | 75% | Left open adrenalectomy, hyperCVAD/  MA, CHOP | Dead (3 mo) |
| 35 | Mizoguchi Y 2005(28) | Japan | 17/M | Bilateral (L 50, R 48) | Adrenal gland, liver, lung, bone marrow, spleen, etc | No | Autopsy | Ⅳ | Yes | 4480 | 34900 | 5.1×10^5^ | N/A | - | N/A | - | N/A | - | N/A | + | + | + | + | N/A | - | Dead (4 days) |
| 36 | Papalkar D 2005(29) | Australia | 41/F | Left (N/A) | Right posterior orbit, lungs, pancreas, spleen, uterus, left adrenal gland, heart, pericardium and meninges | No | Autopsy | Ⅳ | Yes | N/A | N/A | N/A | N/A | + | N/A | N/A | - | - | N/A | + | + | N/A | N/A | N/A | No | Dead (a week) |
| 37 | Seer database (1) | America | 65-69/F | Bilateral(N/A) | Adrenal | N/A | Positive histology | Ⅰ | N/A | N/A | N/A | N/A | N/A | N/A | N/A | N/A | N/A | N/A | N/A | N/A | N/A | N/A | N/A | N/A | Chemotherapy | Dead (less than 1 month) |
| 38 | Seer database (2) | America | 60-64/M | Right(N/A) | Adrenal | N/A | Positive histology | Ⅳ | Yes | N/A | N/A | N/A | N/A | N/A | N/A | N/A | N/A | N/A | N/A | N/A | N/A | N/A | N/A | N/A | N/A | Dead (1 month) |
| 39 | This case | China | 57/M | Left(47) | Left nasal | the left tonsil, the left lobe of the thyroid, left adrenal gland and the left supraclavicular lymph node | Surgical resection | Ⅳ | No | N/A | N/A | N/A | + | N/A | + | - | - | -- | N/A | + | + | + | + | 90% | Radiation, adrenalectomy, P-GEMOX and Sintilimab | Alive (101 mo) |

**Table S3.** The number of SNPs on different regions of the genome and coding regions. CDS, coding sequences; SNP, single nucleotide polymorphism; UTR, untranslated region; ncRNA, noncoding ribonucleic acid.

| **Sample** | **Tumor specimen** | **Adjacent specimen** |
| --- | --- | --- |
| CDS | 19,601 | 20,660 |
| Synonymous_SNP | 10,207 | 10,765 |
| Missense_SNP | 8,995 | 9,467 |
| Stopgain | 63 | 65 |
| Stoploss | 10 | 9 |
| Unknown | 326 | 354 |
| Intronic | 1,172,819 | 1,209,506 |
| UTR3 | 22,982 | 23,684 |
| UTR5 | 4,389 | 4,621 |
| Splicing | 541 | 567 |
| ncRNA_exonic | 8,524 | 9,014 |
| ncRNA_intronic | 134,701 | 138,437 |
| ncRNA_UTR3 | 0 | 0 |
| ncRNA_UTR5 | 0 | 0 |
| ncRNA_splicing | 82 | 88 |
| Upstream | 19,221 | 20,352 |
| Downstream | 20,711 | 21,482 |
| Intergenic | 1,997,101 | 2,056,498 |
| Total | 3,400,005 | 3,504,202 |

**Table S4.** The number of SNPs in different regions of the genome. SNP, single nucleotide polymorphism; Het, heterozygote; Hom, homozygote; TS, transformation; TV, transmutation; dbSNP, the single nucleotide polymorphism.

| **Sample** | **Tumor specimen** | **Adjacent specimen** |
| --- | --- | --- |
| Total | 3,400,005 | 3,504,202 |
| Het | 1,937,075 | 2,016,556 |
| Hom | 1,462,930 | 1,487,646 |
| TS | 2,294,446 | 2,367,529 |
| TV | 1,105,559 | 1,136,673 |
| TS/TV | 2.08 | 2.08 |
| dbSNP percentage | 3362852(98.91%) | 3480778(99.33%) |
| Novel | 37,153 | 23,424 |
| Novel_ TS | 21,032 | 14,392 |
| Novel_TV | 16,121 | 9,032 |
| Novel_TS/TV | 1.30 | 1.59 |

**Table S5.** The number of INDELs on different regions of the genome and coding regions. CDS, coding sequences; SNP, single nucleotide polymorphism; UTR, untranslated region; ncRNA, noncoding ribonucleic acid.

| **Sample** | **Tumor specimen** | **Adjacent specimen** |
| --- | --- | --- |
| CDS | 466 | 488 |
| Frameshift deletion | 64 | 63 |
| Frameshift insertion | 48 | 50 |
| Nonframeshift deletion | 152 | 171 |
| Nonframeshift insertion | 127 | 128 |
| Stopgain | 6 | 5 |
| Stoploss | 0 | 0 |
| Unknown | 69 | 71 |
| Intronic | 180,724 | 193,656 |
| UTR3 | 4,178 | 4,412 |
| UTR5 | 531 | 578 |
| Splicing | 129 | 144 |
| ncRNA_exonic | 895 | 922 |
| ncRNA_intronic | 20,459 | 22,026 |
| ncRNA_UTR3 | 0 | 0 |
| ncRNA_UTR5 | 2 | 2 |
| ncRNA_splicing | 14 | 16 |
| Upstream | 3,379 | 3,667 |
| Downstream | 3,655 | 3,968 |
| Intergenic | 283,079 | 305,446 |
| Total | 497,401 | 535,209 |

**Table S6.** The number of INDELs in different regions of the genome. INDELs, insertions and deletions; Het, heterozygote; Hom, homozygote; dbSNP, the single nucleotide polymorphism.

| **Sample** | **Tumor specimen** | **Adjacent specimen** |
| --- | --- | --- |
| Total | 497,401 | 535,209 |
| Het | 352,840 | 384,603 |
| Hom | 144,561 | 150,606 |
| dbSNP percentage | 442166(88.90%) | 474363(88.63%) |
| Novel | 55,235 | 60,846 |
| Novel_proportion | 11.1% | 11% |

**Table S7.** The number of Somatic SNVs in different regions of the genome. SNVs, single nucleotide variants; CDS, coding sequence; SNP, single nucleotide polymorphism; UTR, untranslated region; ncRNA, noncoding ribonucleic acid.

| **Sample** | **Tumor specimen** |
| --- | --- |
| CDS | 83 |
| Synonymous_SNP | 17 |
| Missense_SNP | 63 |
| Stopgain | 2 |
| Stoploss | 0 |
| Unknown | 1 |
| Intronic | 4841 |
| UTR3 | 66 |
| UTR5 | 17 |
| Splicing | 2 |
| ncRNA_exonic | 23 |
| ncRNA_intronic | 662 |
| ncRNA_UTR3 | 0 |
| ncRNA_UTR5 | 0 |
| ncRNA_splicing | 0 |
| Upstream | 60 |
| Downstream | 65 |
| Intergenic | 10072 |
| Others | 1 |
| Total | 15892 |

**Table S8.** The number of Somatic INDELs in different regions of the genome. INDELs, insertions and deletions; ncRNA, noncoding ribonucleic acid.

| **Sample** | **Tumor specimen** |
| --- | --- |
| CDS | 2 |
| Frameshift_deletion | 1 |
| Frameshift_insertion | 1 |
| Nonframeshift_deletion | 0 |
| Nonframeshift_insertion | 0 |
| Stopgain | 0 |
| Stoploss | 0 |
| Unknown | 0 |
| Intronic | 122 |
| UTR3 | 2 |
| UTR5 | 0 |
| splicing | 0 |
| ncRNA_exonic | 1 |
| ncRNA_intronic | 13 |
| ncRNA_UTR3 | 0 |
| ncRNA_UTR5 | 0 |
| ncRNA_splicing | 0 |
| Upstream | 1 |
| Downstream | 4 |
| Intergenic | 219 |
| Others | 0 |
| Total | 364 |

**Table S9.** Analysis results of predisposing genes. Ref, reference; Alt, alternative; AA, amino acid.

| **Hugo**  **Symbol** | **Chromosome** | **Position** | **Ref**  **allele** | **Alt**  **allele** | **Variant_Classification** | **AAChange** |
| --- | --- | --- | --- | --- | --- | --- |
| HIP1 | 7 | 75192236 | C | A | Splice_Site | NM_001243198:exon11:c.1020+3G>T  NM_005338:exon11:c.1020+3G>T |
| ELF1 | 13 | 41515418 | T | C | Missense_Mutation | ELF1:NM_001145353:exon7:c.A823G:p.I275V  ELF1:NM_172373:exon8:c.A895G:p.I299V |
| ZFHX3 | 16 | 72992900 | C | T | Missense_Mutation | ZFHX3:NM_006885:exon2:c.G1145A:p.G382D |
| AHNAK | 11 | 62295321 | T | C | Missense_Mutation | AHNAK:NM_001620:exon5:c.A6568G:p.N2190D |
| ANK3 | 10 | 61958264 | C | T | Missense_Mutation | ANK3:NM_001204404:exon14:c.G1472A:p.R491Q  ANK3:NM_020987:exon14:c.G1523A:p.R508Q  ANK3:NM_001204403:exon15:c.G1505A:p.R502Q |
| KLF4 | 9 | 110249957 | T | C | Missense_Mutation | KLF4:NM_004235:exon3:c.A718G:p.S240G |
| KLF4 | 9 | 110249959 | C | T | Missense_Mutation | KLF4:NM_004235:exon3:c.G716A:p.G239D |
| PRDM16 | 1 | 3328116 | C | T | Missense_Mutation | PRDM16:NM_022114:exon9:c.C1355T:p.T452M  PRDM16:NM_199454:exon9:c.C1355T:p.T452M |
| RECQL4 | 8 | 145740630 | C | A | Splice_Site | NM_004260:exon8:c.1391-4G>T |
| SMURF2 | 17 | 62568057 | T | C | Missense_Mutation | SMURF2:NM_022739:exon10:c.A875G:p.N292S |
| NSD1 | 5 | 176637769 | T | C | Missense_Mutation | NSD1:NM_022455:exon5:c.T2369C:p.I790T  NSD1:NM_172349:exon6:c.T1562C:p.I521T |
| LAMA2 | 6 | 129837463 | G | T | Missense_Mutation | LAMA2:NM_001079823:exon64:c.G9328T:p.V3110F  LAMA2:NM_000426:exon65:c.G9340T:p.V3114F |
| WWTR1 | 3 | 149245654 | T | G | Missense_Mutation | WWTR1:NM_001168280:exon5:c.A874C:p.T292P  WWTR1:NM_015472:exon5:c.A874C:p.T292P  WWTR1:NM_001168278:exon6:c.A874C:p.T292P |
| EML4 | 2 | 42552650 | A | G | Missense_Mutation | EML4:NM_001145076:exon19:c.A2024G:p.N675S  EML4:NM_019063:exon20:c.A2198G:p.N733S |
| ZNF638 | 2 | 71591314 | G | A | Missense_Mutation | ZNF638:NM_001014972:exon5:c.G1649A:p.R550K  ZNF638:NM_001252612:exon5:c.G1649A:p.R550K  ZNF638:NM_001252613:exon5:c.G1649A:p.R550K  ZNF638:NM_014497:exon5:c.G1649A:p.R550K |
| TRERF1 | 6 | 42237163 | G | T | Missense_Mutation | TRERF1:NM_033502:exon5:c.C166A:p.H56N |
| KMT2C | 7 | 151842355 | T | C | Missense_Mutation | KMT2C:NM_170606:exon54:c.A14057G:p.N4686S |
| ABI1 | 10 | 27066023 | C | T | Missense_Mutation | ABI1:NM_001012750:exon3:c.G433A:p.V145I  ABI1:NM_001012751:exon3:c.G433A:p.V145I  ABI1:NM_001012752:exon3:c.G433A:p.V145I  ABI1:NM_001178119:exon3:c.G433A:p.V145I  ABI1:NM_001178120:exon3:c.G433A:p.V145I  ABI1:NM_001178121:exon3:c.G433A:p.V145I  ABI1:NM_001178122:exon3:c.G433A:p.V145I  ABI1:NM_001178123:exon3:c.G433A:p.V145I  ABI1:NM_001178124:exon3:c.G433A:p.V145I  ABI1:NM_005470:exon3:c.G433A:p.V145I  ABI1:NM_001178116:exon4:c.G484A:p.V162I |
| VTI1A | 10 | 114286892 | T | C | Missense_Mutation | VTI1A:NM_145206:exon4:c.T311C:p.L104P |
| SMO | 7 | 128843429 | C | T | Missense_Mutation | SMO:NM_005631:exon2:c.C536T:p.T179M |
| EIF3E | 8 | 109241340 | T | C | Missense_Mutation | EIF3E:NM_001568:exon6:c.A556G:p.M186V |
| TLX3 | 5 | 170738433 | G | A | Missense_Mutation | TLX3:NM_021025:exon3:c.G706A:p.A236T |
| ZNF384 | 12 | 6798263 | C | G | Splice_Site | NM_133476:exon1:UTR5 |
| SFPQ | 1 | 35658405 | CGGCTGCTGCGGCGGT | C | In_Frame_Del | SFPQ:NM_005066:exon1:c.231_245del:p.77_82del |

**Table S10.** Analysis results of driving genes. Ref, reference; Alt, alternative; AA, amino acid.

| **Hugo**  **Symbol** | **Chromosome** | **Position** | **Ref**  **allele** | **Alt**  **allele** | **Variant_Classification** | **AAChange** |
| --- | --- | --- | --- | --- | --- | --- |
| LEPR | 1 | 66096026 | C | T | Missense_Mutation | LEPR:NM_001198687:exon19:c.C2815T:p.L939F  LEPR:NM_001003680:exon20:c.C2815T:p.L939F |
| ACVR1 | 2 | 158622540 | T | G | Missense_Mutation | ACVR1:NM_001105:exon8:c.A959C:p.H320P  ACVR1:NM_001111067:exon8:c.A959C:p.H320P |
| STAT3 | 17 | 40474482 | T | A | Missense_Mutation | STAT3:NM_003150:exon21:c.A1919T:p.Y640F  STAT3:NM_139276:exon21:c.A1919T:p.Y640F  STAT3:NM_213662:exon21:c.A1919T:p.Y640F |
| TET2 | 4 | 106157174 | A | G | Missense_Mutation | TET2:NM_001127208:exon3:c.A2075G:p.E692G  TET2:NM_017628:exon3:c.A2075G:p.E692G |
| IDH1 | 2 | 209113113 | G | A | Missense_Mutation | IDH1:NM_001282386:exon4:c.C394T:p.R132C  IDH1:NM_001282387:exon4:c.C394T:p.R132C  IDH1:NM_005896:exon4:c.C394T:p.R132C |
| CHD7 | 8 | 61764709 | G | A | Missense_Mutation | CHD7:NM_017780:exon29:c.G5797A:p.A1933T |
| STAT3 | 17 | 40477049 | T | G | Missense_Mutation | STAT3:NM_003150:exon16:c.A1396C:p.N466H  STAT3:NM_139276:exon16:c.A1396C:p.N466H  STAT3:NM_213662:exon16:c.A1396C:p.N466H |
| FAS | 10 | 90773993 | A | G | Missense_Mutation | FAS:NM_152871:exon8:c.A731G:p.D244G  FAS:NM_000043:exon9:c.A794G:p.D265G |
| TP53 | 17 | 7578394 | T | C | Missense_Mutation | TP53:NM_001126115:exon1:c.A140G:p.H47R  TP53:NM_001126116:exon1:c.A140G:p.H47R  TP53:NM_001126117:exon1:c.A140G:p.H47R  TP53:NM_001276697:exon1:c.A59G:p.H20R  TP53:NM_001276698:exon1:c.A59G:p.H20R  TP53:NM_001276699:exon1:c.A59G:p.H20R  TP53:NM_001126118:exon4:c.A419G:p.H140R  TP53:NM_000546:exon5:c.A536G:p.H179R  TP53:NM_001126112:exon5:c.A536G:p.H179R  TP53:NM_001126113:exon5:c.A536G:p.H179R  TP53:NM_001126114:exon5:c.A536G:p.H179R  TP53:NM_001276695:exon5:c.A419G:p.H140R  TP53:NM_001276696:exon5:c.A419G:p.H140R  TP53:NM_001276760:exon5:c.A419G:p.H140R  TP53:NM_001276761:exon5:c.A419G:p.H140R |
| SOS1 | 2 | 39240636 | AAAAG | A | Frame_Shift_Del | SOS1:NM_005633:exon13:c.2128_2131del:p.L710fs |

**References**

1. Mnafe O, Hicham E. Nasal-Type Natural Killer/T-cell Lymphoma With Inaugural Testicular Presentation in a Young Patient. *Cureus* (2024) 16(2):e54733. doi: 10.7759/cureus.54733

2. Liu C, Li F, Mao C, Dangzeng Z, Wang L. Pitfalls in diagnosing a case of extranodal NK/T-cell lymphoma with CD20 aberrant expression and IGH gene rearrangement. *J Cutan Pathol* (2023) 50(12):1052-8. doi: 10.1111/cup.14528

3. Sekar A, Jain S, Bakshi J, Rachagiri S, Bhujade H, Kumar R, et al. Disseminated Nasal subtype Extranodal NK/T-cell lymphoma and its diagnostic difficulties in antemortem biopsies. *Autops Case Rep* (2023) 13:e2023445. doi: 10.4322/acr.2023.445

4. Zhou SL, Wei JG, Li B, Wang XJ, Gong ZQ, Fan PH, et al. [Primary adrenal NK/T cell lymphoma: a clinicopathologic analysis of six cases]. *Zhonghua Bing Li Xue Za Zhi* (2023) 52(7):710-4. doi: 10.3760/cma.j.cn112151-20230120-00060

5. Ji XY, Sheng DP, Yang YQ, Wei YF, Huang X, Liu Q, et al. [Clinical Anslysis of Primary Adrenal NK/T-Cell Lymphoma]. *Zhongguo Shi Yan Xue Ye Xue Za Zhi* (2023) 31(2):396-402. doi: 10.19746/j.cnki.issn.1009-2137.2023.02.013

6. Wu W, Ren K, Li N, Luo Q, Zhou H, Hai T, et al. Central nervous system involvement at initial diagnosis of extranodal NK/T-cell lymphoma: a retrospective study of a consecutive 12-year case series. *Ann Hematol* (2023) 102(4):829-39. doi: 10.1007/s00277-022-05065-6

7. Baba Y, Sakai H, Abe M, Kabasawa N. Extranodal NK/T-cell lymphoma in a unilateral adrenal tumor. *Jpn J Clin Oncol* (2022) 52(12):1450-1. doi: 10.1093/jjco/hyac134

8. Ichikawa S, Saito K, Fukuhara N, Yokoyama H, Onodera K, Onishi Y, et al. Primary adrenal extranodal NK/T-cell lymphoma: A case report and literature review. *Leuk Res Rep* (2020) 14:100223. doi: 10.1016/j.lrr.2020.100223

9. Yu P, Tang T, Tan Y, Wang H, Li Q. Indolent CD30-Positive Extranodal NK/T Cell Lymphoma with Large Cell Transformation: Case Report and Literature Review. *Indian J Otolaryngol Head Neck Surg* (2022) 74(Suppl 2):2376-80. doi: 10.1007/s12070-020-02190-4

10. Dong P, Wang L, Shen G, Li L. Primary adrenal extranasal NK/T cell lymphoma with subcutaneous involvement demonstrated on FDG PET/CT: A clinical case report. *Medicine (Baltimore)* (2019) 98(11):e14818. doi: 10.1097/MD.0000000000014818

11. Han Y, Kim KH, Choi IH. Cytological findings of NK/T-cell lymphoma in pericardial effusion: A case report with a review of the literature. *Indian J Pathol Microbiol* (2019) 62(3):473-6. doi: 10.4103/IJPM.IJPM_540_18

12. Huang Y, Shi X, Zhong P, Wang Y, Xiao H, Zhou X, et al. De Novo Testicular Extranodal NK/T-Cell Lymphoma: A Clinicopathologic Study of 21 Cases With Review of Additional 18 Cases in the Literature. *Am J Surg Pathol* (2019) 43(4):549-58. doi: 10.1097/PAS.0000000000001210

13. Huang J, Huang DP, Yang YQ, Jin XK, Pan HS. [Analysis of Clinical Characteristics for Primary Adrenal Lymphoma]. *Zhongguo Shi Yan Xue Ye Xue Za Zhi* (2018) 26(4):1067-71. doi: 10.7534/j.issn.1009-2137.2018.04.021

14. Hu L, Xu W, Wang M, Wang P, Han G, Lin C. A case report of primary unilateral adrenal NK/T cell lymphoma: good clinical outcome with trimodality treatment. *BMC Cancer* (2017) 17(1):15. doi: 10.1186/s12885-016-3019-1

15. Wang X, Gong Z, Li SX, Yan W, Song Y. Extranodal nasal-type natural killer/T-cell lymphoma with penile involvement: a case report and review of the literature. *BMC Urol* (2017) 17(1):77. doi: 10.1186/s12894-017-0273-8

16. Kabnurkar R, Agrawal A, Epari S, Purandare N, Shah S, Rangarajan V. Unilateral primary adrenal natural killer/T-cell lymphoma: Role of fluorine-18 fluorodeoxyglucose positron emission tomography/computed tomography for staging and interim response assessment. *Indian J Nucl Med* (2016) 31(1):52-4. doi: 10.4103/0972-3919.172363

17. Tian C, Wang Y, Zhu L, Yu Y, Zhang Y. Primary bone natural killer/T cell lymphoma, nasal type without EBV infection: a case report. *Int J Clin Exp Pathol* (2015) 8(11):14836-9.

18. Liang DN, Yang ZR, Wang WY, Zhao S, Yang QP, Tang Y, et al. Extranodal nasal type natural killer/T-cell lymphoma of testis: report of seven cases with review of literature. *Leuk Lymphoma* (2012) 53(6):1117-23. doi: 10.3109/10428194.2011.645209

19. Poli M, Sève P, Merrot O, Grange JD, Kodjikian L. Diagnosis of a multicentric extranodal nasal-type natural killer T-cell lymphoma made with an anterior chamber tap. *Retin Cases Brief Rep* (2012) 6(1):46-9. doi: 10.1097/ICB.0b013e3182051e0b

20. Tsukahara T, Takasawa A, Murata M, Okumura K, Nakayama M, Sato N, et al. NK/T-cell lymphoma of bilateral adrenal glands in a patient with pyothorax. *Diagn Pathol* (2012) 7:114. doi: 10.1186/1746-1596-7-114

21. Ishihara S, Kano O, Ikeda K, Shimokawa R, Kawabe K, Iwasaki Y. Clinicoradiological changes of brain NK/T cell lymphoma manifesting pure akinesia: a case report. *BMC Neurol* (2011) 11:137. doi: 10.1186/1471-2377-11-137

22. Kang SM, Kim WJ, Lee KA, Baek HS, Park TS, Jin HY. Mainly adrenal gland involving NK/T-cell nasal type lymphoma diagnosed with delay due to mimicking adrenal hemorrhage. *J Korean Med Sci* (2011) 26(10):1386-90. doi: 10.3346/jkms.2011.26.10.1386

23. Nagireddy S, Avery RA, Kakivayi S, Matin K. Natural killer T-cell lymphoma presenting as adrenal mass and partial adrenal insufficiency followed by CNS involvement. *J Clin Oncol* (2011) 29(15):e431-3. doi: 10.1200/JCO.2010.33.6396

24. Dunning KK, Wudhikarn K, Safo AO, Holman CJ, McKenna RW, Pambuccian SE. Adrenal extranodal NK/T-cell lymphoma diagnosed by fine-needle aspiration and cerebrospinal fluid cytology and immunophenotyping: a case report. *Diagn Cytopathol* (2009) 37(9):686-95. doi: 10.1002/dc.21077

25. Mozos A, Ye H, Chuang WY, Chu JS, Huang WT, Chen HK, et al. Most primary adrenal lymphomas are diffuse large B-cell lymphomas with non-germinal center B-cell phenotype, BCL6 gene rearrangement and poor prognosis. *Mod Pathol* (2009) 22(9):1210-7. doi: 10.1038/modpathol.2009.87

26. Toba A, Tamura Y, Osajima Y, Kinbara Y, Sato M, Yamaga R, et al. [A case of nasal NK/T cell lymphoma presenting with bilateral giant adrenal tumors]. *Nihon Ronen Igakkai Zasshi* (2008) 45(6):660-5. doi: 10.3143/geriatrics.45.660

27. Thompson MA, Habra MA, Routbort MJ, Holsinger FC, Perrier ND, Waguespack SG, et al. Primary adrenal natural killer/T-cell nasal type lymphoma: first case report in adults. *Am J Hematol* (2007) 82(4):299-303. doi: 10.1002/ajh.20811

28. Mizoguchi Y, Nakamura K, Miyagawa S, Nishimura S, Arihiro K, Kobayashi M. A case of adolescent primary adrenal natural killer cell lymphoma. *Int J Hematol* (2005) 81(4):330-4. doi: 10.1532/IJH97.04143

29. Papalkar D, Sharma S, Francis IC, Downie JA, Thanakrishnan G, Hughes LJ. A rapidly fatal case of T-cell lymphoma presenting as idiopathic orbital inflammation. *Orbit* (2005) 24(2):131-3. doi: 10.1080/01676830490916064

**Supplementary Figure**


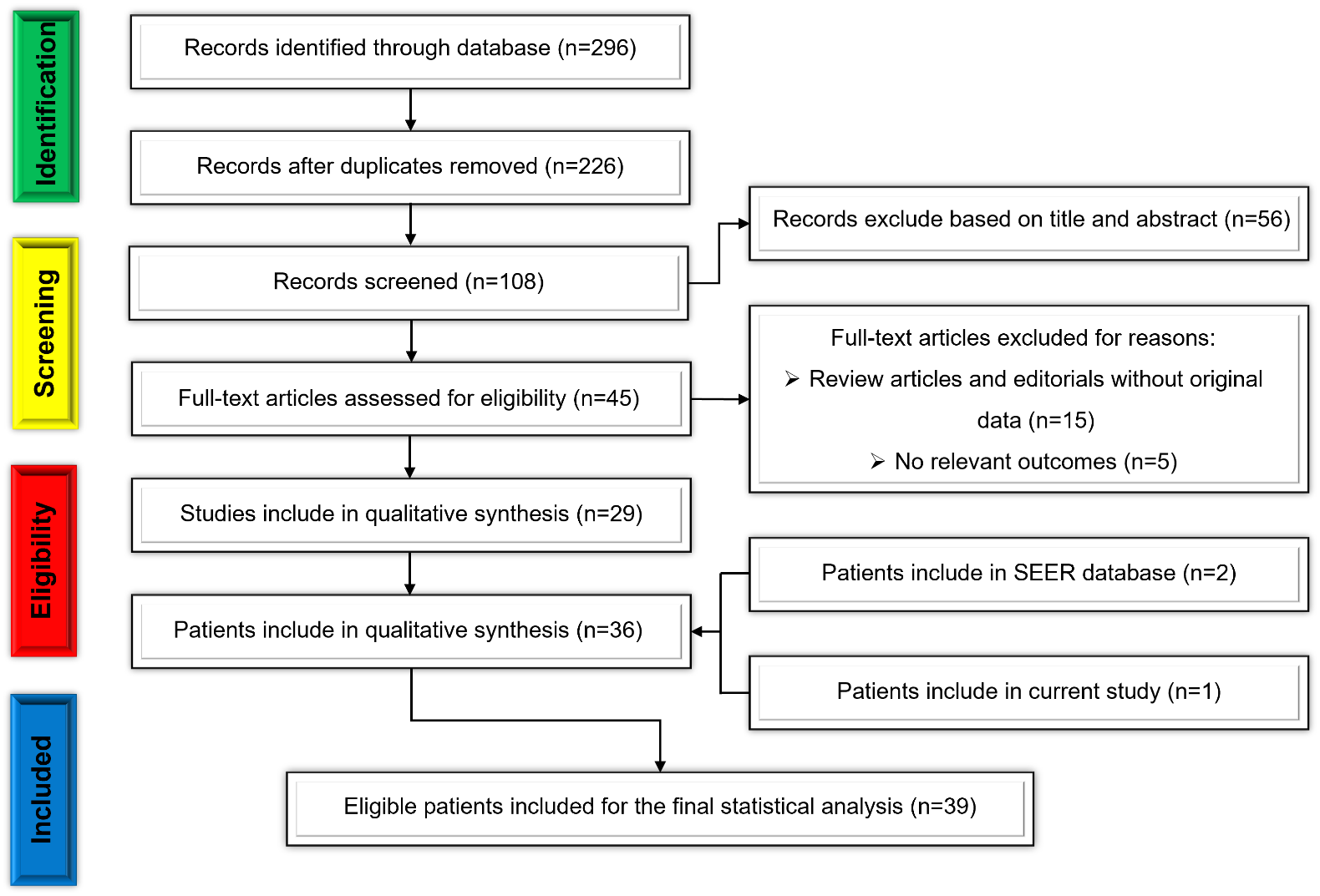


**Supplementary Figure 1.** Flow chart of the selection process for patients with adrenal extra-nodal natural killer/T -cell lymphoma.
